# Gut metabolites and inflammation factors in non-alcoholic fatty liver disease: A systematic review and meta-analysis

**DOI:** 10.1038/s41598-020-65051-8

**Published:** 2020-06-01

**Authors:** Xiongfeng Pan, Shi Wu Wen, Atipatsa C. Kaminga, Aizhong Liu

**Affiliations:** 1 Department of Epidemiology and Health Statistics, Xiangya School of Public Health, Central South University, Changsha, China; 2 OMNI Research Group, Ottawa Hospital Research Institute, Ottawa, Canada; 3 Department of Obstetrics and Gynaecology and School of Epidemiology and Public Health, University of Ottawa Faculty of Medicine, Ottawa, Ontario Canada; 4 Department of Mathematics and Statistics, Mzuzu University, Mzuzu, Malawi

**Keywords:** Diagnostic markers, Non-alcoholic fatty liver disease

## Abstract

The interaction of gut microbiota, related metabolites and inflammation factors with nonalcoholic fatty liver disease (NAFLD) remains unclearly defined. The aim of this systematic review and meta-analysis was to synthesize previous study findings to better understand this interaction. Relevant research articles published not later than September, 2019 were searched in the following databases: Web of Science, PubMed, Embase, and Cochrane Library. The search strategy and inclusion criteria for this study yielded a total of 47 studies, of which only 11 were eligible for meta-analysis. The narrative analysis of these articles found that there is interplay between the key gut microbiota, related metabolites and inflammation factors, which modulate the development and progression of NAFLD. In addition, the results of meta-analysis showed that probiotic supplementation significantly decreased tumor necrosis factor-α (TNF-α) in NAFLD patients (standardized mean difference (SMD) = −0.52, confidence interval (CI): −0.86 to −0.18, and *p* = 0.003) and C-reactive protein (CRP) (SMD = −0.62, CI: −0.80 to −0.43, and *p* < 0.001). However, whether therapies can target TNF-α and CRP in order treat NAFLD still needs further investigation. Therefore, these results suggest that the interaction of the key gut microbiota, related metabolites and inflammation factors with NAFLD may provide a novel therapeutic target for the clinical and pharmacological treatment of NAFLD.

## Introduction

Non-alcoholic fatty liver disease (NAFLD), the leading chronic liver disease in the world, has become a growing public health problem due to its steadily rising prevalence in recent years as well as possibility of ending into cirrhosis and hepatocellular carcinoma^1,2^. Noteworthy, lifestyle interventions remain first-line treatments for NAFLD. For example, dietary and physical exercises interventions have been shown to improve transaminase and insulin sensitivity, and reduce body mass index in well-designed trials^3^. Nevertheless, it must be acknowledged that dietary and physical exercises interventions are difficult to be sustained in daily life, possibly because of lack of motivation, genetic background, adaptation of basal metabolic rates, failure to lose weight and hormonal disorders^4^. In addition, a Cochrane network meta-analysis indicated that the effect of drugs (including the thiazolidinediones, insulin sensors and antioxidants, etc.) in the treatment of NAFLD is very uncertain^5^. Therefore, effective therapy for NAFLD is not available thus far, which has spurred multidisciplinary research to better appreciate the potential intricate pathogenesis of NAFLD^6^.

For example, a recent “multi-hit” hypothesis proposed that gut–liver axis (GLA) dysfunction (i.e., bacterial overgrowth, alteration of mucosa permeability, intestinal dysbiosis) may play a key role in promoting the molecular mechanism of NAFLD and triggering the development of non-alcoholic fatty liver (NAFL) with simple steatosis to non-alcoholic steatohepatitis (NASH)^7^.

Besides, in several human studies, it was suggested that there has been a significant difference in the abundance of gut microbiota between NAFLD patients and healthy controls. That is, unlike in the healthy controls, the dysbiosis of the gut microbiota in the NAFLD patients initiated the immune homeostasis^8^. In this regard, the gut microbiota were carried to the liver through the portal vein, leading to the over-activation of immune cells in the liver^9^. Moreover, gut-derived pro-inflammatory metabolites such as peptidoglycan, lipoteichoic acid and lipopolysaccharide activate the signal pathways of inflammatory cytokines in the liver which in turn lead to severe inflammation, fibrosis and liver damage in NAFLD^10^.

Currently, it is generally accepted that inflammation is also the main factor contributing to the pathogenesis and progression of NAFLD and liver injury. Also, some evidence has suggested that different resident liver cell types interact with the gut microbiota system and its metabolites, and this interaction activate uncontrolled immune responses in the NAFLD^11^. Furthermore, gut microbiota and related metabolites may trigger the production of a cascade of cytokines, help perpetuate adverse inflammatory responses, and lead to the release of a variety of inflammatory markers^12^. In addition, various serum markers of inflammation would be produced in the adverse inflammatory response of the liver, including interleukins (ILs), tumor necrosis factor (TNF), C-reactive protein (CRP) and other general immunity markers^13^. Therefore, it could be suggested that gut microbiota, its related metabolites and inflammation factors in NAFLD may be potential targets for pharmacological and clinical treatment of NAFLD.

Note that meta-analysis is widely used in statistical analysis to examine data from a number of independent studies on the same subject in order to determine overall trends. Thus, meta-analysis found its practical application in many disciplines, including educational psychology and biomarker exploration^14,15^. Although previous reviews have discussed the relationship between NAFLD and gut microbiota and its related metabolites or inflammation factors, the interaction between gut microbiota, its related metabolites and inflammation factors in NAFLD is unknown^10,13,16^. Therefore, the aim of this study was to synthesize the findings of studies on the interaction of gut microbiota, its related metabolites and inflammation factors with NAFLD using systematic review and meta-analysis.

## Results

### Study selection

**Figure 1:**
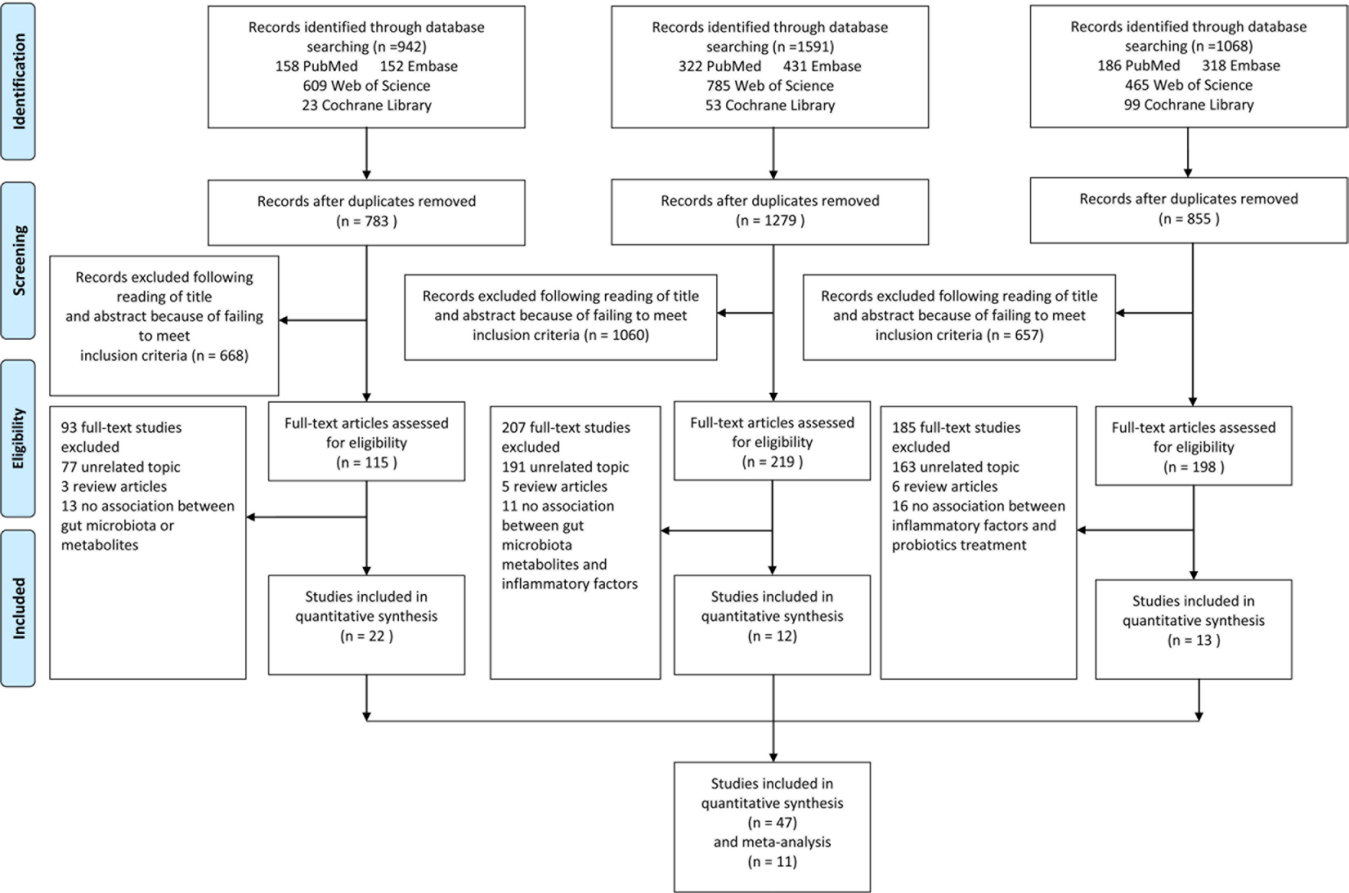
Flowchart of study selection. Showing the process by which relevant studies were retrieved from the databases, assessed, and selected, or excluded. Preferred reporting items for systematic reviews and meta-analyses (PRISMA) diagram for study search.

Figure 1 shows the study selection flow chart. The search strategy in the databases identified a total of 3,601 articles. Title and abstract screening resulted in 3,069 articles excluded. The full texts of the remaining 532 articles were reviewed with respect to the study selection criteria, and this process excluded 485 articles. Therefore, 47 studies were included in this systematic review and meta-analysis.

### Study characteristics

The characteristics of the 47 eligible studies are summarized in Appendices 2–4, and in Table 1. Specifically, 10 studies were included to demonstrate gut microbiota in NAFLD and 12 studies to demonstrate gut microbial metabolites in NAFLD (Appendices 2, 3). In addition, gut microbiota in NAFLD has been described in terms of its phylum, family, and genus. Meanwhile, the different roles played by different gut microbial metabolites in NAFLD have been described as well as the main results of the corresponding studies. Furthermore, the relationship between gut metabolites and inflammatory factors in NAFLD, and the main results including different gut metabolites that would induce different inflammatory factors in NAFLD, have been described in Table 2. Moreover, in relation to the NAFLD and its inflammatory factors after probiotics treatment, a detailed description of the characteristics (including type of study, sample size, country, mean age, follow up time, NAFLD type, and diagnosis of NAFLD) of eligible studies exploring inflammatory factors and probiotics treatment has been provided in Appendix. Accordingly, the main outcomes of NAFLD and its inflammatory factors after probiotics treatment are described in Table 1.

**Table 1:** NAFLD and its inflammatory factors after probiotics treatment.

**Study**	**Material**	**Outcome**	**Results**
Aller 2011^39^	L. bulgaricus and S.thermophilus	IL-6, TNF	TNF alpha and IL-6 remained unchanged after treatment
Asgharian 2016^40^	L. acidophilus, L. casei, L. rhamnosus, L. bulgaricus, B. breve, B. longum, S.thermophiles þ FOS	hs-CRP	CRP values remained static in either group.
Ekhlasi 2017^41^	Syniotic (L. casei, L. rhamnosus, S.thermophilus, B. breve,L. acidophilus, B. longum, L. bulgaricus þ FOS)	TNF	After 8 weeks of intervention, combined symbiotic and alpha-tocopherol, symbiotic and alpha-tocopherol alone administration, compared with the placebo, resulted in significant decreases in SBP, serum MDA, serum TNFα concentrations. A significant decrease in serum AST, ALT and ALP was seen.
Eslamparast 2014^42^	Lactobacillus casei, Lactobacillus rhamnosus, Staphylococcus thermophilus, Bifidobacterium breve, Lactobacillus acidophilus, Bifidobacterium longum,	NF-κB and reduction of TNF-α	Inhibition of NF-κB and reduction of TNF-α
Loguercio 2002^43^	Lactobacillus acidophilus, Bifidobacterium bifidum, Lactobacillus rhamnosus, Lactobacillus plantarum, Lactobacillus salivarius, Lactobacillus bulgaricus, Lactobacillus casei, Bifidobacterium lactis,	ALT, GGT, and TNF-α	NASH patients: decreased ALT, GGT, and TNF-α.
Malaguarnera 2012^44^	Bifidobacterium longum and FOS	AST, CRP, TNF-α and endotoxin	NASH patients:Decreased AST, CRP, TNF-α and endotoxin
Mofidi 2017^45^	Symbiotic (seven strains (L. casei, L. rhamnosus, S.thermophilus, B. breve, L. acidophilus, B. longum and L. bulgaricus) and fructooligosaccharide)	hs-CRP, TNF	Furthermore, serum levels of fasting blood sugar, TAG and most of the inflammatory mediators reduced in the synbiotic group significantly compared with the placebo group.
Mykhal’chyshyn 2013^46^	Lactobacillus, Lactococcus, Propionibacterium, Bifidobacterium, Aceticbacterium	IL-6, IL-8, TNF-α, IL-1β and IFN-α	Decreased IL-6, IL-8, TNF-α, IL-1β and IFN-α.
Sepideh 2016^47^	Lactobacillus casei, Lactobacillus acidophilus, Lactobacillus rhamnosus, Lactobacillus bulgaricus, Bifidobacterium breve, Bifidobacterium longum, and Staphylococcus thermophilus	FBS, insulin, IR, TNF-α, and IL-6	Decreased FBS, insulin, IR, TNF-α, and IL-6.
Sherf-Dagan 2018^48^	L. acidophilus, B. bifidum, L. rhamnosus, Lactococcuslactis, L. casei, B. breve, S.thermophiles, B. longum, L. paracasei, L. plantarum, B. infatis	hs-CRP, IL-6, TNF, IL-10	Fibrosis, liver-enzymes, CRP, leptin and cytokeratin-18 levels were significantly reduced in the probiotics NAFLD groups
Vajro 2011^49^	Lactobacillus rhamnosus GG	ALT,TNF-α	Decreased ALT and TNF-α
Wang 2018^50^	Bifidobacterium, Bacillus, Enterococcus	ALT, AST, TNF-α	Decreased in TNF-α
Yang 2012^51^	Bacillus subtilis and Enterococcus	TNF-α,IL-6,ALT	Decreased in TNF-α,IL-6

NAFLD, Nonalcoholic fatty liver disease; NASH, non-alcoholic steatohepatitis; ALT, Alanine aminotransferase; AST, Aspartate aminotransferase; CRP, C-reactive protein; IL-6, interleukin 6; IL-8, interleukin 8; IL-10, interleukin 10; TC, total cholesterol; FBS, fasting blood sugar; DBP, diastolic blood pressure; SBP, systolic blood pressure; TG, Triglycerides; LDL-C, low density lipoprotein cholesterol; HDL-C, high-density lipoprotein cholesterol; TNF-α: Tumor Necrosis Factor.

**Table 2:** Gut metabolites and inflammatory factors in NAFLD.

**Study**	**Material**	**Result**
D’Mello 2015^52^	Endotoxins	The metabolites of the gut microbiota, including endotoxins, activate the inflammatory response in the liver when they cannot be cleared by kuppfer cells
Ruiz 2007^53^	LPS, LBP	Elevated serum LBP levels and TNF-α overexpression were observed in NAFLD and NASH patients, and the serum LBP levels and TNF-α expression were higher in NASH patients than in NAFLD patients
Liu 2014^54^	Endotoxins	Activation of the endotoxins TLR4 signaling pathway significantly increases the release of a series of inflammatory cytokines, including TNF-α, IL-1β, IL-6 and IL-12, and participates in multiple steps of the development and progression of NAFLD
Leoni 2018^55^	Endotoxins	Dysregulation of proinflammatory cytokines and adipokines is almost universally present in NAFLD patients, which directly or indirectly (mainly through the TLR4 signaling pathway) lead to hepatocyte injury. In addition, oxidative stress and hepatocyte apoptosis are associated with the progression of NASH
Chavez-Talavera 2017^56^	BA	BAs regulate the metabolism and inflammation through FXR and Takeda G-protein receptor 5, which possess the function of controlling the metabolism of BAs, lipids and carbohydrates, and regulating the expression of inflammatory genes
Janssen 2017^57^	BA	FXR is able to activate small heterodimer partner to reduce the expression of sterol regulatory element-binding protein 1, which is a major regulator in new fat formation; inhibition of FXR(FXR, farnesoid X receptor) leads to the abnormal lipid metabolism and development of NAFLD
Zhang 2016^58^	BA,	Hereditary obesity, insulin resistance and NAFLD may be prevented or reversed by glycine-β-muricholic acid, an intestinal FXR antagonist, which possesses the ability to change the intestinal bacterial composition.
Fukunishi 2014^59^	LPS	Activation of TLR4 induced by LPS results in the secretion of inflammatory cytokines (e.g.,IL-6,IL-1β,and TNF-α) and chemokines from Kupffer cells, leadingtohepaticdamage and NASH.
Kawasaki 2008^60^	Peptidoglycan (PGN)	The sub-structures of PGN, such as meso-diaminopimelic acid PGN (meso-DAP PGN) and muramyl dipeptide PGN (MDP PGN), can mediate the generation of pro-inflammatory cytokines through nuclear factor-κB(NF-κB)/mitogen-activated protein kinase (MAPK) dependent activation of NOD1 (Nucleotide Binding Oligomerization Domain Containing 1) and NOD2 (Nucleotide Binding Oligomerization Domain Containing 2).
Gomes 2016^61^	Bacterial DNAs	Bacterial DNAs play a vital role in the progression of NASH by the direct activation of immune cells including macrophages, NK cells, B cells, and dendritic cells. The sensing of bacterial DNA by TLR9 in immune cells initiates the activation of NF-κB/MAPK, followed by the secretion of IL-12 and TNF-α
Natividad 2018^62^	Indole-3-acetic acid (IAA)	IAA dose dependently reduces the induction of pro-inflammatory cytokines including TNF-α, MCP-1, and IL-1β by LPS, leading to a reduction in the synthesis of FFAs and palmitate in macrophage cell line. Besides, IAA alleviates the lipogenesis mediated by cytokine and free fatty acids via its direct action on hepatocytes in an AhR-dependent manner. The evidences above suggest a protective role of IAA against NAFLD through acting on both macrophages and hepatocytes.
Ma 2006^63^	CA and BA	FXR activation by cholic acid (CA) reduces glucose levels by inhibiting expression of multiple genes related to gluconeogenesis in the liver. Aside from FXR, Takeda-G-protein-receptor-5 (TGR5) is another classic receptor for bile acids. In hepatic tissue, TGR5 is expressed in Kupffer and endothelial cells and functions to modulate liver inflammation and glucose metabolism, and to improve insulin sensitivity. TGR5 mitigates inflammatory response through the inhibition of NF-κB signaling and cytokines generation in macrophages.

NAFLD, Nonalcoholic fatty liver disease; NASH, non-alcoholic steatohepatitis; LPS, lipopolysaccharides; LBP, Lipopolysaccharide-binding protein; SCFAs, Short-chain fatty acids; BA, bile acids; CA, Cholic acid; CRP, C-reactive protein; IL-6, interleukin 6; IL-8, interleukin 8; IL-10, interleukin 10; TC, total cholesterol; TG, Triglycerides; LDL-C, low density lipoprotein cholesterol; HDL-C, high-density lipoprotein cholesterol; TNF-α: Tumor Necrosis Factor.

### Main outcomes

#### Changes in gut microbiota of NAFLD

**Figure 2:**
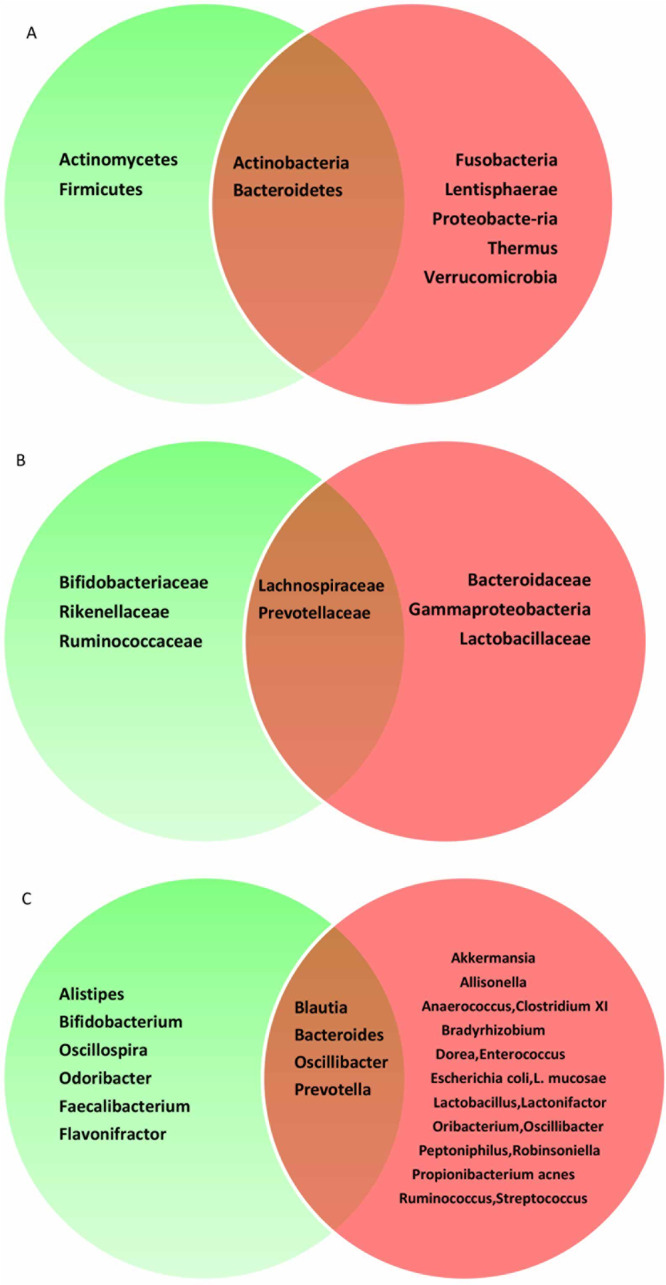
Venn diagram of different gut microbiota between the NAFLD and the control group. The green area represents a decrease in gut microbiota in NAFLD group compared to the control group. The red areas represent elevated gut microbiota in NAFLD group compared to the control group. The middle part represents the contradiction in the current study. A, Phylum; B, Family;C, Genus; NAFLD, nonalcoholic fatty liver disease.

In Fig. 2, the differences between NAFLD patients and the control group at the levels of phylum, family and genus of gut microbiota are presented. In this regard, at the level of phylum, the relative abundance of Actinomycetes and Firmicutes phyla were increased in NAFLD. On the contrary, the phyla, Fusobacteria, Lentisphaerae, proteobacte-ria, Thermus and Verrucomicrobia, were decreased in the NAFLD patients unlike in the control group. However, the difference in the abundance of the phyla, Actinobacteria and Bacteroidetes between the NAFLD patients and the controls was contradictory. Furthermore, at the family level, the combination of a low abundance of the families, Bifidobacteriaceae, Rikenellaceae and Ruminococcaceae, and a high abundance of the key families, Bacteroidaceae, Gammaproteobacteria, and Lactobacillaceae, represented the abnormal state of the gut microbiota in NAFLD. Nevertheless, results of the families, Lachnospiraceae and Prevotellaceae, were contradictory. Considering the gut microbiota genus, NAFLD caused changes in numerous gut microbiota genus such that the Alistipes, Bifidobacterium, Oscillospira, Odoribacter, Faecalibacterium and Flavonifractor, which reduced the abundance of the gut microbiota, whereas the relative abundance of Akkermansia, Allisonella, Anaerococcus, Clostridium XI, Bradyrhizobium, Dorea, Enterococcus, Escherichia coli, L. mucosae, Lactobacillus, Lactonifactor, Oribacterium, Oscillibacter, Peptoniphilus, Robinsoniella, Propionibacterium acnes, Ruminococcus and Streptococcus was increased. Nevertheless, the results of the genera, Blautia, Bacteroides, Oscillibacter and Prevotella were inconclusive.

#### Gut metabolites and inflammatory factors in NAFLD

The gut bioactive metabolites include the bacterial DNAs, endotoxins, lipopolysaccharides, peptidoglycan and extracellular vesicles, which are derived from the gut microbiota as well as the metabolites such as short-chain fatty acids (SCFAs), branched-chain amino acids, bile acids, cholic acid, indole, butyrate, propionate, endogenous ethanol and trimethylamine-N-oxide (TMAO). Appendix shows the details.

Table 2 summarizes the association between gut metabolites and inflammatory factors in modulating the pathological process of NAFLD. Among them, endotoxin and lipopolysaccharide increase the release of a range of inflammatory factors including IL-6, IL-12, IL-1β and TNF-α in the development of NAFLD. Additionally, bile acids and cholic acids not only had direct antibacterial properties, but also they mitigated inflammatory response through the inhibition of nuclear factor kappa-B (NF-κB) signaling and cytokines generation in macrophages. Meanwhile, peptidoglycan from gram-positive bacteria promoted inflammatory response. Bacterial DNA played an important role in activating immune cells such as NK cells, dendritic cells, macrophages, B cells and macrophages, followed by the secretion of IL-12 and TNF-α. Studies have also shown that indole-3-acetic acid dose-dependently reduced lipopolysaccharide-induced proinflammatory cytokines, including MCP-1, IL-1β, and TNF-α.

#### The effects of probiotic therapies on inflammatory factors in NAFLD

**Figure 3:**
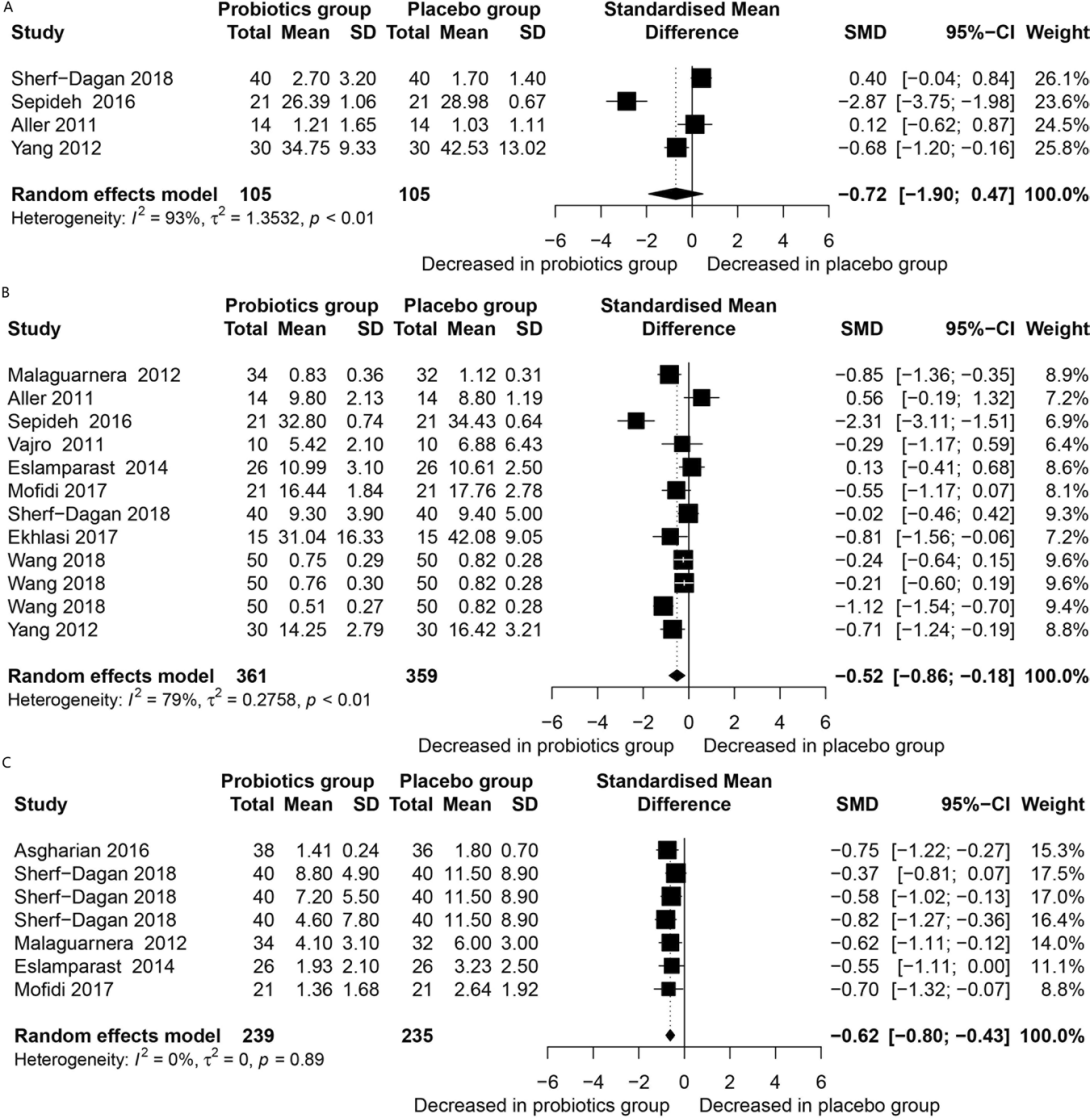
Forest plot of inflammation factors between probiotics group and placebo group. Study effect sizes of IL-6, TNF-α and CRP concentration differences between NAFLD and controls. Each data marker represents a study, and the size of the data marker is proportional to the total number of individuals in that study. The summary effect size for each IL-6, TNF-α and CRP concentration is denoted by a diamond. NAFLD, nonalcoholic fatty liver disease; SMD, standardized mean difference. A, IL-6; B, TNF-α; C, CRP.

Table 1 presents the NAFLD and its association with inflammatory factors after probiotics treatment, while Fig. 3 presents forest plots of the results of the inflammatory factors and their association with NAFLD between the probiotics group and placebo group. It was observed that probiotics treatment (without additional intervention) decreased CRP (SMD = −0.62, CI − 0.80 to −0.43, *p* < 0.001), with no heterogeneity (*I²* = 0); and levels of TNF-α (SMD = −0.52, CI − 0.86 to −0.18, *p* = 0.003) but with considerable heterogeneity (*I²* = 79%). However, the two groups did not differ significantly with respect to IL-6 concentrations. Sensitivity analysis showed that there was a slight change in the SMD and its corresponding 95% CI when each study was removed in turn, indicating that the current meta-analysis data were relatively stable. Furthermore, only studies that included the TNF-α group were eligible for publication bias assessment, which was done by Egger funnel plot and Egger’s test (Appendix). Accordingly, there was no significant publication bias detected (t = −0.61, df =10, *p* = 0.5539). Moreover, the results of risk of bias were, A, B, and C, implying that the risk of bias was negligible (Appendix).

## Discussion

This study has revealed that there may be an interaction of gut microbiota, related metabolites and inflammation factors with NAFLD. In addition, this study assessed the efficacy of probiotic therapies in modifying inflammation factors in NAFLD.

As regard to gut microbiota and gut metabolites, although gut microbiota of humans was highly variable, this review has shown that patients with NAFLD reported more harmful bacteria (such as Gammaproteobacteria) and less beneficial bacteria (such as Bifidobacteriaceae) than the control group^17^. Considering several contradictory findings of related studies with similar designs (such as Lachnospiraceae and Prevotellaceae), additional large-scale studies on the gut bioactive metabolites and metagenomics characteristics of gut microbiota in NAFLD are needed to confirm these results^16^.

It has long been known that high-fat diet could induce gut dysbiosis, and prolonged gut dysbiosis could produce different gut metabolites, including trimethylamine, SCFAs, glucagon-like peptide 1, lipopolysaccharide, free fatty acids, free cholesterol, and ethanol^10^. In addition, these gut metabolites may promote systemic inflammatory response, such as lipopolysaccharide, which can activate Kupffer cells to produce TNF-α and cause insulin resistance^13^. Thus, insulin resistance increases the accumulation of hepatic triglycerides by promoting triglyceride synthesis, hepatic uptake of free fatty acids and peripheral lipolysis in NAFLD^7^. Therefore, patients affected by NAFLD may have a gut dysbiosis, accompanied by small intestine bacterial overgrowth and elevation of gut metabolites such as endotoxin. Noteworthy, increased intestinal permeability may lead to increased bacterial migration and, under this condition, gut metabolites and some bacteria may enter the portal circulation by permeating the intestinal barrier and reach the liver^12,18^. Clinical studies have also shown that almost all patients with NAFLD had abnormal levels of inflammatory cytokines, which triggered the inflammatory response pathway of the gut microflora in NAFLD. Furthermore, experimental studies have shown that cytokine-mediated oxidative stress, mitochondria and endoplasmic reticulum dysfunction may promote the development of steatohepatitis from simple steatosis via the toll-like receptor signaling pathway^12,19^.

**Figure 4:**
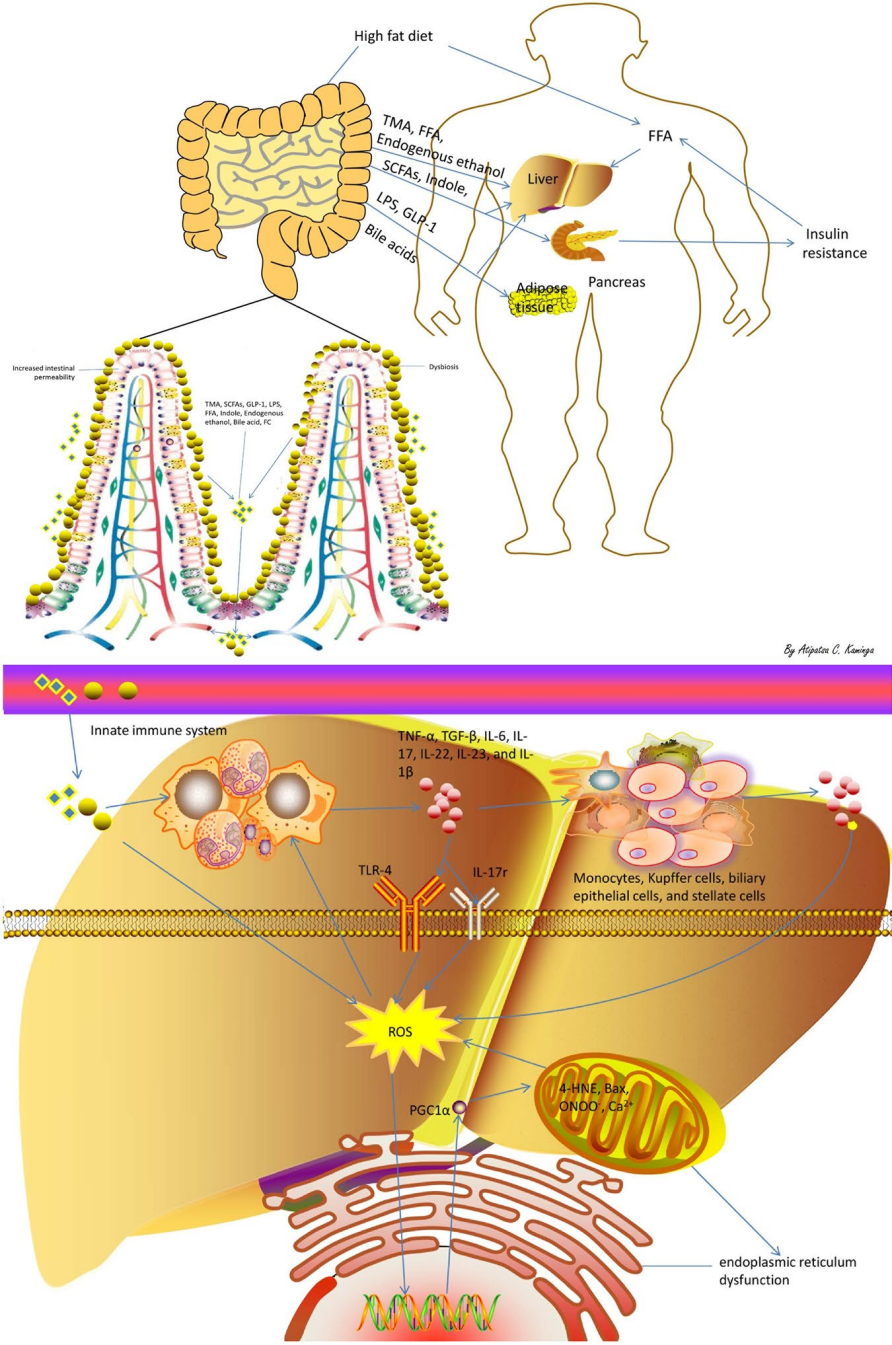
Summarizes the hypothesis mechanism process of gut microbiota, related metabolites and inflammation factors on NAFLD pathophysiology. TMA, Trimethylamine; SCFAs, Short-chain fatty acids; GLP-1, glucagon-like peptide 1; LPS, lipopolysaccharide; FFA, free fatty acids; FC, free cholesterol; ROS, Reactive oxygen species; 4-HNE, 4-hydroxy-2-nonenal; FMO3: Flavin-containing monooxygenase 3; TMAO: Trimethylamine-N-oxide; IL-1/−6/-17/-22/-23, interleukin-1/-6/-17/-22/-23; TNF-α, tumour necrosis factor alpha; TLR-4, Toll-like receptor 4; IL-17r, interleukin-17 receptors.

Accumulated evidence suggested that oxidative stress played an important role in the multi-hit hypothesis model of progression of NAFLD^18^. At the organelles level, mitochondria and endoplasmic reticulum are the main sites of ROS formation, producing pro-apoptotic peroxidized lipids (4-hydroxy-2-nonenal) and Bax, and promoting the synthesis of respiratory proteins through PGC1α as well as the transfer of Ca^2+^ and ONOO^20,21^. Consequently, these may promote activation of stellate cells, macrophages, and Kupffer cells, which further promote endoplasmic reticulum and mitochondrial dysfunction, hence inducing inflammation, fibrosis and apoptosis in NAFLD^12^. Therefore, these results supported the hypothesis that interactions among the liver, the immune system and the gut microflora related metabolites have a key role in the development of NASH from simple steatosis. An illustration of this hypothesis is shown in Fig. 4 (by KA).

Considering the gut metabolites and inflammatory factors in NAFLD, accumulating evidence has found that gut microbiota communities generate a variety of metabolites, which are bioactive substances that interact with the host liver cells in NAFLD^13^. Among them, endotoxin and lipopolysaccharide are active metabolites of gram-negative bacterial envelope components, and significantly increase the (mainly through activation of TLR4 signaling pathway) release of a range of inflammatory factors, including IL-6, IL-12, IL-1β and TNF-α in the development of NAFLD^22^. Additionally, exposure of the liver to endotoxin and lipopolysaccharide causes pathological and metabolic changes that induce an acute inflammatory response and early accumulation of inflammatory cells, such as monocytes, neutrophils and lymphocytes. Moreover, inflammatory cells release proteases, other enzymes and reactive oxygen metabolites, leading to pathological progression of liver injury in NAFLD.

Furthermore, bile acids are regulators of lipid, glucose metabolism, and modulate inflammation in NAFLD. Therefore, the gut microbiota converts the primary bile acids (including chenodeoxycholic acid and cholic acid at the colon and distal small intestine) to secondary bile acids, such as ursodeoxycholic acidursodeoxycholic acid, lithocholic acid, and deoxycholic acid^23^. Also, bile acids can regulate the composition of gut microbiota, which in turn can regulate the capacity and composition of bile acids^24^. Thus, the destruction of bile acid-flora interaction can promote the development of NAFLD inflammation^25^. Meanwhile, studies have shown that farnesoid X receptor (FXR) plays an important role in bile acid-flora interactions. Therefore, bile acids are the ligands for the FXR, a member of a family of nuclear hormone receptors, widely found in the ileum and liver that regulate a variety of metabolic pathways^26^. The regulation of FXR signal has become a potential target for the prevention and treatment of NAFLD and related metabolic disorders^26^. Furthermore, not only do bile acids and cholic acids have direct antibacterial properties, but also mitigate inflammatory response through the inhibition of NF-κB signaling and cytokines generation in macrophages, mainly by activating Takeda-G-protein-receptor-5^27^. Meanwhile, peptidoglycan from gram-positive bacteria promotes inflammatory response, primarily through the activation of mitogen-activated protein kinase (MAPK)/NF-κB of the mediated proinflammatory cytokine generation. Subsequently, it also plays an important role in activating immune cells such as NK cells, dendritic cells, macrophages, B cells and macrophages. In addition, bacterial DNA activates MAPK/NF-κB (mainly through activation of TLR9 signaling pathway)^28^.

It is noteworthy that the hepatic steatosis is considered to be the benign beginning of the NAFLD, which is reversible without serious liver injury at this stage. Therefore, the preceding discussion in relation to the results of this study clarifies the roles of gut microbiota, related metabolites and inflammation factors in the pathophysiological process of pure steatosis of liver to NASH. It is hoped that this evidence may provide new insights for future researches and development of drugs targeted at preventing the hepatic steatosis progression to NASH, steatohepatitis-related associated cirrhosis and hepatocellular carcinoma. Interestingly, a recent meta-analysis also showed that probiotics reduced liver transaminase, total cholesterol, TNF-α, and insulin resistance in NAFLD patients^29^. However, that meta-analysis analyzed studies which considered only one inflammatory factor, TNF-α. In this regard, that meta-analysis did not investigate other inflammatory factor levels with respect to the association between probiotics and NAFLD, which has been addressed in this study.

Therefore, this study has shown that there is an indirect evidence of the effects of the probiotic (beneficial gut microbiota) interventions on inflammation and liver damage, which indicated significant reductions in inflammatory factors (CRP and TNF-α) in NAFLD after probiotics were used. Nevertheless, there was no statistically significant difference in the concentrations of IL-6 between the NAFLD patients and the controls, which may be attributed to the small sample sizes involving IL-6. Therefore, large-scale clinical studies on this phenomenon are needed in the future.

Additionally, a systematic review suggested that pentoxifylline, an anti-TNF-α agent, directly targets the inflammatory process. In this regard, reduced AST and ALT levels may improve liver histological scores in patients with NALFD/NASH^30^. However, anti-TNF-α therapy for NAFLD may have significant side effects. Therefore, large, prospective and well-designed randomized controlled studies are needed to explore medications with better efficacy for NAFLD. Besides, new therapeutic targets for inflammatory signaling pathways are also worth exploring, as well as the treatment of NAFLD with CRP as the target.

Also, in agreement with the findings of this study, some studies have shown that activated carbon could reduce metabolites in NAFLD. For example, in addition to the probiotic supplementation, Yaq-001 (Yaqrit Ltd.) carefully designed and developed a novel synthetic (both macroporous and microporous) activated carbon for treating gut microbiota and related metabolites modulation in NAFLD^31^. In this regard, it has been shown that Yaq-001 (when administered orally) could reduce the transintestinal migration of gut microbiota and related metabolites, such as dimethylarginine, ammonia, bacteria-derived products, acetaldehyde, hydrophobic bile acids, and inflammation factors, including TNF-α and IL-6^31^. A safety and efficacy study on Yaq-001 is being conducted through the European Commission Horizon program. A clinical trial is also under way (NCT03962608). Future studies should focus on developing more specific novel therapeutics for NAFLD based on bioengineering technology with high efficacy and specificity. For example, utilizing targeted engineered individual microbiota instead of fecal transplants, or utilizing engineered microbiota capable of producing anti-inflammatory or antioxidants molecules to replace the antibiotics, or utilizing engineered compounds that modulate or adsorb the gut microbiota-derived metabolites of interest.

There are some limitations of this study, which should be acknowledged. Firstly, the heterogeneity was very high in the meta-analysis of TNF-α. Given that the subjects in this study came from different countries and ethnic groups, this heterogeneity may be due to the large individual differences in TNF-α concentrations, especially considering the significant side effects of TNF-α in the pathogenesis of NAFLD, hence the findings on TNF-α in this study should be interpreted with caution. Secondly, this study only analyzed the overall level of each study and did not quantify the important clinical indicators of individual patient level (also due to the lack of information in the original study), such as race, smoking, alcohol drinking and blood pressure, which could also be a reason for the large heterogeneity. Thirdly, the probiotic intervention was a well-designed experiment, and whether it is possible to replicate the results in the general population is unknown. Therefore, large population cohort studies are needed in the future to provide a causal relationship between intestinal flora and its metabolites and NAFLD.

Although there is currently no efficient treatment available for NAFLD, the findings of this systematic review/meta-analysis suggest that the gut microbiota, related metabolites and inflammation factors (especially CRP and TNF-α) network presently appear to be innovative and potentially promising treatment targets. Future studies should focus on developing more specific novel therapeutics for NAFLD based on bioengineering technology with high efficacy and specificity.

## Methods

This systematic review and meta-analysis was conducted according to the Cochrane handbook 5.1.0, and the results were reported in accordance with the Preferred Reporting Items for Systematic Reviews and Meta-Analyses (PRISRM)^32^.

### Search strategy

Relevant articles published no later than September, 2019 were searched in the following databases: Web of Science, PubMed, Embase, and Cochrane Library. Experienced librarians of Central South University, China, helped in formulating search strategies using keywords (Appendix). The search outcomes were screened by two investigators, AK and XP, to identify potential relevant studies for this systematic review and meta-analysis.

### Eligibility criteria

The inclusion criteria for selecting eligible studies were as follows: (1) studies reported the criteria for the diagnosis of NAFLD; (2) studies provided a measure of association between gut microbiota and metabolites in NAFLD; (3) studies provided a measure of association between gut microbiota and inflammatory factors in NAFLD; (4) studies provided a measure of association between inflammatory factors and probiotics treatment in NAFLD; (5) studies were peer-reviewed publication; and (6) studies were published in English. On the contrary, studies were excluded if they: (1) were case reports, letter or reviews; (2) reported NAFLD in combination with other diseases; and (3) had the antibiotic or other gut microbiota pharmacologically challenged before their measurements. Two researchers [KA and SW] independently identified the eligible studies, and their discrepancies were resolved by involving the third researcher [AL].

### Data extraction and preparation

A standardized data extraction form was developed to record data of each eligible study in connection with the aims of this systematic review and meta-analysis. Therefore, data related to the following variables were extracted from each eligible study: (1) first author’s name and year of publication; (2) subject’s characteristics such as NAFLD or NASH; (3) characteristics of gut microbiota such as phylum, family, and genus; (4) characteristics of gut microbiota and its metabolites; (5) characteristics of gut metabolites and inflammatory factors; and (6) characteristics of inflammatory factors after probiotics treatment. Two researchers [KA and SW] independently extracted data from each study by using EpiData 3.0 and Microsoft Excel 2010. Discrepancies in this activity were resolved by [AL]. Data for the risk of bias assessment for the randomized studies was extracted using the Cochrane Collaboration’s tool by two researchers [KA and SW] who also used Egger’s test to assess the significance of the bias^33^.

### Statistical analysis

Random effects model was used to assess the differences in concentrations of the inflammatory factors in NAFLD between the probiotic group and the placebo group. This model was chosen because it is suitable when synthesizing findings of studies with varying populations and criteria used to define outcomes, which was the case with the eligible studies for this meta-analysis. The R software (version R 3.5.2) was used to perform meta-analysis in the R packages, ‘meta’ and ‘metafor’. Specifically, the group differences in the concentrations of the inflammatory factors in NAFLD were measured in terms of standardized mean difference (SMD), calculated as Cohen’s d, and corresponding 95% confidence intervals (CI)^34^. A high effect size was represented by SMD > 0.8, whereas 0.5 ≤ SMD ≤ 0.8 and SMD < 0.5 represented moderate and low effect sizes, respectively. In addition, heterogeneity between-study was assessed using the Cochrane Q statistic and the level of heterogeneity was measured by the *I²* statistic^35,36^. Thus, low, moderate and high heterogeneity was represented by the *I²* statistic of less than 25%, 25~75% and greater than 75%, respectively^37^. Moreover, sensitivity analysis was repeated every time each study was omitted in turn^38^. All the statistical tests were two-sided and performed at the 5% significance level. In addition to meta-analysis, a narrative description was performed for: gut microbiota’s phylum, family, and genus in NAFLD; the different roles played by different gut microbial metabolites in NAFLD; the relationship between gut metabolites and inflammatory factors in NAFLD; and different gut metabolites that would induce different inflammatory factors in NAFLD.

## Supplementary information

Appendix.
