# Supplementary material for: Gut metabolites and inflammation factors in non-alcoholic fatty liver disease: A systematic review and meta-analysis
Source: Sci Rep. 2020 Jun 1;10:8848. doi: 10.1038/s41598-020-65051-8 (PMC7264254; doi:10.1038/s41598-020-65051-8)
Supplement: Supplementary file 1 — Appendix. [file 41598_2020_65051_MOESM1_ESM.doc]

**Gut metabolites and inflammation factors in non-alcoholic fatty liver disease: A systematic review and meta-analysis**

Xiongfeng Pan^1^, Shi Wu Wen^2,3^, Atipatsa C. Kaminga^1, 4^, Aizhong Liu^1*^

^1^Xiangya School of Public Health, Central South University, Changsha, China.

^2^OMNI Research Group, Ottawa Hospital Research Institute;

^3^Department of Obstetrics and Gynaecology and School of Epidemiology and Public Health, University of Ottawa Faculty of Medicine, Ottawa, Ontario, Canada.

^4^Department of Mathematics and Statistics, Mzuzu University, Mzuzu, Malawi.

^*^Correspondence to: Aizhong Liu PhD

Department of Epidemiology and Health Statistics, Xiangya School of Public Health, Central South University.

110 Xiangya Rd, Changsha, Hunan, P.R. of China 410078

Tel: 86-135-17310050

Fax: 86-731-84805454

E-mail: lazroy@live.cn

**Supplementary appendix**

**Contents**

**Appendix 1a**: Electronic search strategies for gut microbiota or metabolites

**Appendix 1b**: Electronic search strategies for gut microbiota and inflammatory factors

**Appendix 1c**: Electronic search strategies for inflammatory factors and probiotics treatment

**Appendix 2**: Gut microbiota in NAFLD

**Appendix 3**: Gut microbial metabolites in NAFLD

**Appendix 4**: Characteristics of included studies for inflammatory factors and probiotics treatment

**Appendix 5**: Egger funnel plot for the TNF-α in NAFLD group

**Appendix 6**: Risk of bias assessments

**Appendix 1a**

158 of PubMed

 (((Gastrointestinal Microbiome[Title/Abstract] OR Gastrointestinal Microbiomes[Title/Abstract] OR Microbiome, Gastrointestinal[Title/Abstract] OR Gut Microbiome[Title/Abstract] OR Gut Microbiomes[Title/Abstract] OR Microbiome, Gut[Title/Abstract] OR Gut Microflora[Title/Abstract] OR Microflora, Gut[Title/Abstract] OR Gut Microbiota[Title/Abstract] OR Gut Microbiotas[Title/Abstract] OR Microbiota, Gut[Title/Abstract] OR Gastrointestinal Flora[Title/Abstract] OR Flora, Gastrointestinal[Title/Abstract] OR Gut Flora[Title/Abstract] OR Flora, Gut[Title/Abstract] OR Gastrointestinal Microbiota[Title/Abstract] OR Gastrointestinal Microbiotas[Title/Abstract] OR Microbiota, Gastrointestinal[Title/Abstract] OR Gastrointestinal Microbial Community[Title/Abstract] OR Gastrointestinal Microbial Communities[Title/Abstract] OR Microbial Community, Gastrointestinal[Title/Abstract] OR Gastrointestinal Microflora[Title/Abstract] OR Microflora, Gastrointestinal[Title/Abstract] OR Gastric Microbiome[Title/Abstract] OR Gastric Microbiomes[Title/Abstract] OR Microbiome, Gastric[Title/Abstract] OR Intestinal Microbiome[Title/Abstract] OR Intestinal Microbiomes[Title/Abstract] OR Microbiome, Intestinal[Title/Abstract] OR Intestinal Microbiota[Title/Abstract] OR Intestinal Microbiotas[Title/Abstract] OR Microbiota, Intestinal[Title/Abstract] OR Intestinal Microflora[Title/Abstract] OR Microflora, Intestinal[Title/Abstract] OR Intestinal Flora[Title/Abstract] OR Flora, Intestinal[Title/Abstract] OR Enteric Bacteria[Title/Abstract] OR Bacteria, Enteric[Title/Abstract])) AND (Metabolites[Title/Abstract] OR Metabolic Pathways[Title/Abstract] OR Metabolic Pathway[Title/Abstract] OR Pathway, Metabolic[Title/Abstract] OR Pathways, Metabolic[Title/Abstract] OR Metabolic Networks[Title/Abstract] OR Metabolic Network[Title/Abstract] OR Network, Metabolic[Title/Abstract] OR Networks, Metabolic[Title/Abstract] OR Metabolomics[Title/Abstract] OR Metabolomic[Title/Abstract] OR Metabonomics[Title/Abstract] OR Metabonomic[Title/Abstract] OR Metabolome[Title/Abstract] OR Metabolomes[Title/Abstract] OR Metabolic Profile[Title/Abstract] OR Metabolic Profiles[Title/Abstract] OR Profile, Metabolic[Title/Abstract] OR Profiles, Metabolic[Title/Abstract])) AND (Non alcoholic Fatty Liver Disease[Title/Abstract] OR NAFLD[Title/Abstract] OR Nonalcoholic Fatty Liver Disease[Title/Abstract] OR Fatty Liver, Nonalcoholic[Title/Abstract] OR Fatty Livers, Nonalcoholic[Title/Abstract] OR Liver, Nonalcoholic Fatty[Title/Abstract] OR Livers, Nonalcoholic Fatty[Title/Abstract] OR Nonalcoholic Fatty Liver[Title/Abstract] OR Nonalcoholic Fatty Livers[Title/Abstract] OR Nonalcoholic Steatohepatitis[Title/Abstract] OR Nonalcoholic Steatohepatitides[Title/Abstract] OR Steatohepatitides, Nonalcoholic[Title/Abstract] OR Steatohepatitis, Nonalcoholic[Title/Abstract] OR Non-alcoholic Fatty Liver Disease[Title/Abstract])

152 of Embase

('gastrointestinal microbiome':ab,ti OR 'gastrointestinal microbiomes':ab,ti OR 'microbiome, gastrointestinal':ab,ti OR 'gut microbiome':ab,ti OR 'gut microbiomes':ab,ti OR 'microbiome, gut':ab,ti OR 'gut microflora':ab,ti OR 'microflora, gut':ab,ti OR 'gut microbiota':ab,ti OR 'gut microbiotas':ab,ti OR 'microbiota, gut':ab,ti OR 'gastrointestinal flora':ab,ti OR 'flora, gastrointestinal':ab,ti OR 'gut flora':ab,ti OR 'flora, gut':ab,ti OR 'gastrointestinal microbiota':ab,ti OR 'gastrointestinal microbiotas':ab,ti OR 'microbiota, gastrointestinal':ab,ti OR 'gastrointestinal microbial community':ab,ti OR 'gastrointestinal microbial communities':ab,ti OR 'microbial community, gastrointestinal':ab,ti OR 'gastrointestinal microflora':ab,ti OR 'microflora, gastrointestinal':ab,ti OR 'gastric microbiome':ab,ti OR 'gastric microbiomes':ab,ti OR 'microbiome, gastric':ab,ti OR 'intestinal microbiome':ab,ti OR 'intestinal microbiomes':ab,ti OR 'microbiome, intestinal':ab,ti OR 'intestinal microbiota':ab,ti OR 'intestinal microbiotas':ab,ti OR 'microbiota, intestinal':ab,ti OR 'intestinal microflora':ab,ti OR 'microflora, intestinal':ab,ti OR 'intestinal flora':ab,ti OR 'flora, intestinal':ab,ti OR 'enteric bacteria':ab,ti OR 'bacteria, enteric':ab,ti) AND ('non alcoholic fatty liver disease':ab,ti OR nafld:ab,ti OR 'nonalcoholic fatty liver disease':ab,ti OR 'fatty liver, nonalcoholic':ab,ti OR 'fatty livers, nonalcoholic':ab,ti OR 'liver, nonalcoholic fatty':ab,ti OR 'livers, nonalcoholic fatty':ab,ti OR 'nonalcoholic fatty liver':ab,ti OR 'nonalcoholic fatty livers':ab,ti OR 'nonalcoholic steatohepatitis':ab,ti OR 'nonalcoholic steatohepatitides':ab,ti OR 'steatohepatitides, nonalcoholic':ab,ti OR 'steatohepatitis, nonalcoholic':ab,ti OR 'non-alcoholic fatty liver disease':ab,ti) AND (metabolites:ab,ti OR 'metabolic pathways':ab,ti OR 'metabolic pathway':ab,ti OR 'pathway, metabolic':ab,ti OR 'pathways, metabolic':ab,ti OR 'metabolic networks':ab,ti OR 'metabolic network':ab,ti OR 'network, metabolic':ab,ti OR 'networks, metabolic':ab,ti OR metabolomics:ab,ti OR metabolomic:ab,ti OR metabonomics:ab,ti OR metabonomic:ab,ti OR metabolome:ab,ti OR metabolomes:ab,ti OR 'metabolic profile':ab,ti OR 'metabolic profiles':ab,ti OR 'profile, metabolic':ab,ti OR 'profiles, metabolic':ab,ti)

609 of Web of Science

(Metabolites OR Metabolic Pathways OR Metabolic Pathway OR Pathway, Metabolic OR Pathways, Metabolic OR Metabolic Networks OR Metabolic Network OR Network, Metabolic OR Networks, Metabolic OR Metabolomics OR Metabolomic OR Metabonomics OR Metabonomic OR Metabolome OR Metabolomes OR Metabolic Profile OR Metabolic Profiles OR Profile, Metabolic OR Profiles, Metabolic) AND TOPIC: (Gastrointestinal Microbiome OR Gastrointestinal Microbiomes OR Microbiome, Gastrointestinal OR Gut Microbiome OR Gut Microbiomes OR Microbiome, Gut OR Gut Microflora OR Microflora, Gut OR Gut Microbiota OR Gut Microbiotas OR Microbiota, Gut OR Gastrointestinal Flora OR Flora, Gastrointestinal OR Gut Flora OR Flora, Gut OR Gastrointestinal Microbiota OR Gastrointestinal Microbiotas OR Microbiota, Gastrointestinal OR Gastrointestinal Microbial Community OR Gastrointestinal Microbial Communities OR Microbial Community, Gastrointestinal OR Gastrointestinal Microflora OR Microflora, Gastrointestinal OR Gastric Microbiome OR Gastric Microbiomes OR Microbiome, Gastric OR Intestinal Microbiome OR Intestinal Microbiomes OR Microbiome, Intestinal OR Intestinal Microbiota OR Intestinal Microbiotas OR Microbiota, Intestinal OR Intestinal Microflora OR Microflora, Intestinal OR Intestinal Flora OR Flora, Intestinal OR Enteric Bacteria OR Bacteria, Enteric) AND TOPIC: (Non alcoholic Fatty Liver Disease OR NAFLD OR Nonalcoholic Fatty Liver Disease OR Fatty Liver, Nonalcoholic OR Fatty Livers, Nonalcoholic OR Liver, Nonalcoholic Fatty OR Livers, Nonalcoholic Fatty OR Nonalcoholic Fatty Liver OR Nonalcoholic Fatty Livers OR Nonalcoholic Steatohepatitis OR Nonalcoholic Steatohepatitides OR Steatohepatitides, Nonalcoholic OR Steatohepatitis, Nonalcoholic OR Non-alcoholic Fatty Liver Disease)

23 of Cochrane

Non alcoholic Fatty Liver Disease OR NAFLD OR Nonalcoholic Fatty Liver Disease OR Fatty Liver, Nonalcoholic OR Fatty Livers, Nonalcoholic OR Liver, Nonalcoholic Fatty OR Livers, Nonalcoholic Fatty OR Nonalcoholic Fatty Liver OR Nonalcoholic Fatty Livers OR Nonalcoholic Steatohepatitis OR Nonalcoholic Steatohepatitides OR Steatohepatitides, Nonalcoholic OR Steatohepatitis, Nonalcoholic OR Non-alcoholic Fatty Liver Disease in Title Abstract Keyword AND Metabolites OR Metabolic Pathways OR Metabolic Pathway OR Pathway, Metabolic OR Pathways, Metabolic OR Metabolic Networks OR Metabolic Network OR Network, Metabolic OR Networks, Metabolic OR Metabolomics OR Metabolomic OR Metabonomics OR Metabonomic OR Metabolome OR Metabolomes OR Metabolic Profile OR Metabolic Profiles OR Profile, Metabolic OR Profiles, Metabolic in Title Abstract Keyword AND Gastrointestinal Microbiome OR Gastrointestinal Microbiomes OR Microbiome, Gastrointestinal OR Gut Microbiome OR Gut Microbiomes OR Microbiome, Gut OR Gut Microflora OR Microflora, Gut OR Gut Microbiota OR Gut Microbiotas OR Microbiota, Gut OR Gastrointestinal Flora OR Flora, Gastrointestinal OR Gut Flora OR Flora, Gut OR Gastrointestinal Microbiota OR Gastrointestinal Microbiotas OR Microbiota, Gastrointestinal OR Gastrointestinal Microbial Community OR Gastrointestinal Microbial Communities OR Microbial Community, Gastrointestinal OR Gastrointestinal Microflora OR Microflora, Gastrointestinal OR Gastric Microbiome OR Gastric Microbiomes OR Microbiome, Gastric OR Intestinal Microbiome OR Intestinal Microbiomes OR Microbiome, Intestinal OR Intestinal Microbiota OR Intestinal Microbiotas OR Microbiota, Intestinal OR Intestinal Microflora OR Microflora, Intestinal OR Intestinal Flora OR Flora, Intestinal OR Enteric Bacteria OR Bacteria, Enteric in Title Abstract Keyword

**Appendix 1b**

322 of PubMed

(((((Non alcoholic Fatty Liver Disease[Title/Abstract] OR NAFLD[Title/Abstract] OR Nonalcoholic Fatty Liver Disease[Title/Abstract] OR Fatty Liver, Nonalcoholic[Title/Abstract] OR Fatty Livers, Nonalcoholic[Title/Abstract] OR Liver, Nonalcoholic Fatty[Title/Abstract] OR Livers, Nonalcoholic Fatty[Title/Abstract] OR Nonalcoholic Fatty Liver[Title/Abstract] OR Nonalcoholic Fatty Livers[Title/Abstract] OR Nonalcoholic Steatohepatitis[Title/Abstract] OR Nonalcoholic Steatohepatitides[Title/Abstract] OR Steatohepatitides, Nonalcoholic[Title/Abstract] OR Steatohepatitis, Nonalcoholic[Title/Abstract] OR Non-alcoholic Fatty Liver Disease[Title/Abstract]))) AND (Gastrointestinal Microbiome[Title/Abstract] OR Gastrointestinal Microbiomes[Title/Abstract] OR Microbiome, Gastrointestinal[Title/Abstract] OR Gut Microbiome[Title/Abstract] OR Gut Microbiomes[Title/Abstract] OR Microbiome, Gut[Title/Abstract] OR Gut Microflora[Title/Abstract] OR Microflora, Gut[Title/Abstract] OR Gut Microbiota[Title/Abstract] OR Gut Microbiotas[Title/Abstract] OR Microbiota, Gut[Title/Abstract] OR Gastrointestinal Flora[Title/Abstract] OR Flora, Gastrointestinal[Title/Abstract] OR Gut Flora[Title/Abstract] OR Flora, Gut[Title/Abstract] OR Gastrointestinal Microbiota[Title/Abstract] OR Gastrointestinal Microbiotas[Title/Abstract] OR Microbiota, Gastrointestinal[Title/Abstract] OR Gastrointestinal Microbial Community[Title/Abstract] OR Gastrointestinal Microbial Communities[Title/Abstract] OR Microbial Community, Gastrointestinal[Title/Abstract] OR Gastrointestinal Microflora[Title/Abstract] OR Microflora, Gastrointestinal[Title/Abstract] OR Gastric Microbiome[Title/Abstract] OR Gastric Microbiomes[Title/Abstract] OR Microbiome, Gastric[Title/Abstract] OR Intestinal Microbiome[Title/Abstract] OR Intestinal Microbiomes[Title/Abstract] OR Microbiome, Intestinal[Title/Abstract] OR Intestinal Microbiota[Title/Abstract] OR Intestinal Microbiotas[Title/Abstract] OR Microbiota, Intestinal[Title/Abstract] OR Intestinal Microflora[Title/Abstract] OR Microflora, Intestinal[Title/Abstract] OR Intestinal Flora[Title/Abstract] OR Flora, Intestinal[Title/Abstract] OR Enteric Bacteria[Title/Abstract] OR Bacteria, Enteric[Title/Abstract])) AND (Inflammation*[Title/Abstract] OR Immune Activation[Title/Abstract] OR Interleukin*[Title/Abstract] OR Cytokine*[Title/Abstract] OR Interferon[Title/Abstract] OR Lymphocyte[Title/Abstract] OR Macrophage[Title/Abstract] OR Microglia[Title/Abstract] OR Tumor Necrosis Factor-alpha[Title/Abstract] OR C-Reactive Protein[Title/Abstract] OR Transforming growth factor[Title/Abstract] OR IFN[Title/Abstract] OR IL[Title/Abstract] OR CRP[Title/Abstract] OR TGF[Title/Abstract] OR TNF[Title/Abstract] OR Inflammatory factor[Title/Abstract] OR pro-inflammatory cytokine[Title/Abstract] OR chemokine[Title/Abstract] OR inflammatory cytokine[Title/Abstract] OR YKL[Title/Abstract] OR nuclear factor kappa[Title/Abstract] OR hypoxia inducible factor[Title/Abstract] OR hs-CRP[Title/Abstract] OR interleukin-1 receptor antagonist[Title/Abstract] OR E-selectin[Title/Abstract] OR monocyte chemoattractant protein[Title/Abstract] OR MCP[Title/Abstract] OR intercellular adhesion molecule[Title/Abstract] OR ICAM[Title/Abstract] OR Interleukin receptor antagonist[Title/Abstract] OR soluble adhesion molecules[Title/Abstract] OR Chemokines[Title/Abstract]))

431of Embase

('gastrointestinal microbiome':ab,ti OR 'gastrointestinal microbiomes':ab,ti OR 'microbiome, gastrointestinal':ab,ti OR 'gut microbiome':ab,ti OR 'gut microbiomes':ab,ti OR 'microbiome, gut':ab,ti OR 'gut microflora':ab,ti OR 'microflora, gut':ab,ti OR 'gut microbiota':ab,ti OR 'gut microbiotas':ab,ti OR 'microbiota, gut':ab,ti OR 'gastrointestinal flora':ab,ti OR 'flora, gastrointestinal':ab,ti OR 'gut flora':ab,ti OR 'flora, gut':ab,ti OR 'gastrointestinal microbiota':ab,ti OR 'gastrointestinal microbiotas':ab,ti OR 'microbiota, gastrointestinal':ab,ti OR 'gastrointestinal microbial community':ab,ti OR 'gastrointestinal microbial communities':ab,ti OR 'microbial community, gastrointestinal':ab,ti OR 'gastrointestinal microflora':ab,ti OR 'microflora, gastrointestinal':ab,ti OR 'gastric microbiome':ab,ti OR 'gastric microbiomes':ab,ti OR 'microbiome, gastric':ab,ti OR 'intestinal microbiome':ab,ti OR 'intestinal microbiomes':ab,ti OR 'microbiome, intestinal':ab,ti OR 'intestinal microbiota':ab,ti OR 'intestinal microbiotas':ab,ti OR 'microbiota, intestinal':ab,ti OR 'intestinal microflora':ab,ti OR 'microflora, intestinal':ab,ti OR 'intestinal flora':ab,ti OR 'flora, intestinal':ab,ti OR 'enteric bacteria':ab,ti OR 'bacteria, enteric':ab,ti) AND ('non alcoholic fatty liver disease':ab,ti OR nafld:ab,ti OR 'nonalcoholic fatty liver disease':ab,ti OR 'fatty liver, nonalcoholic':ab,ti OR 'fatty livers, nonalcoholic':ab,ti OR 'liver, nonalcoholic fatty':ab,ti OR 'livers, nonalcoholic fatty':ab,ti OR 'nonalcoholic fatty liver':ab,ti OR 'nonalcoholic fatty livers':ab,ti OR 'nonalcoholic steatohepatitis':ab,ti OR 'nonalcoholic steatohepatitides':ab,ti OR 'steatohepatitides, nonalcoholic':ab,ti OR 'steatohepatitis, nonalcoholic':ab,ti OR 'non-alcoholic fatty liver disease':ab,ti) AND (inflammation*:ab,ti OR 'immune activation':ab,ti OR interleukin*:ab,ti OR cytokine*:ab,ti OR interferon:ab,ti OR lymphocyte:ab,ti OR macrophage:ab,ti OR microglia:ab,ti OR 'tumor necrosis factor-alpha':ab,ti OR 'c-reactive protein':ab,ti OR 'transforming growth factor':ab,ti OR ifn:ab,ti OR il:ab,ti OR crp:ab,ti OR tgf:ab,ti OR tnf:ab,ti OR 'inflammatory factor':ab,ti OR 'pro-inflammatory cytokine':ab,ti OR chemokine:ab,ti OR 'inflammatory cytokine':ab,ti OR ykl:ab,ti OR 'nuclear factor kappa':ab,ti OR 'hypoxia inducible factor':ab,ti OR 'hs crp':ab,ti OR 'interleukin-1 receptor antagonist':ab,ti OR 'e selectin':ab,ti OR 'monocyte chemoattractant protein':ab,ti OR mcp:ab,ti OR 'intercellular adhesion molecule':ab,ti OR icam:ab,ti OR 'interleukin receptor antagonist':ab,ti OR 'soluble adhesion molecules':ab,ti OR chemokines:ab,ti)

785 of Web of Science

(Inflammation* OR Immune Activation OR Interleukin* OR Cytokine* OR Interferon OR Lymphocyte OR Macrophage OR Microglia OR Tumor Necrosis Factor-alpha OR C-Reactive Protein OR Transforming growth factor OR IFN OR IL OR CRP OR TGF OR TNF OR Inflammatory factor OR pro-inflammatory cytokine OR chemokine OR inflammatory cytokine OR YKL OR nuclear factor kappa OR hypoxia inducible factor OR hs-CRP OR interleukin-1 receptor antagonist OR E-selectin OR monocyte chemoattractant protein OR MCP OR intercellular adhesion molecule OR ICAM OR Interleukin receptor antagonist OR soluble adhesion molecules OR Chemokines) AND TOPIC: (Gastrointestinal Microbiome OR Gastrointestinal Microbiomes OR Microbiome, Gastrointestinal OR Gut Microbiome OR Gut Microbiomes OR Microbiome, Gut OR Gut Microflora OR Microflora, Gut OR Gut Microbiota OR Gut Microbiotas OR Microbiota, Gut OR Gastrointestinal Flora OR Flora, Gastrointestinal OR Gut Flora OR Flora, Gut OR Gastrointestinal Microbiota OR Gastrointestinal Microbiotas OR Microbiota, Gastrointestinal OR Gastrointestinal Microbial Community OR Gastrointestinal Microbial Communities OR Microbial Community, Gastrointestinal OR Gastrointestinal Microflora OR Microflora, Gastrointestinal OR Gastric Microbiome OR Gastric Microbiomes OR Microbiome, Gastric OR Intestinal Microbiome OR Intestinal Microbiomes OR Microbiome, Intestinal OR Intestinal Microbiota OR Intestinal Microbiotas OR Microbiota, Intestinal OR Intestinal Microflora OR Microflora, Intestinal OR Intestinal Flora OR Flora, Intestinal OR Enteric Bacteria OR Bacteria, Enteric) AND TOPIC: (Non alcoholic Fatty Liver Disease OR NAFLD OR Nonalcoholic Fatty Liver Disease OR Fatty Liver, Nonalcoholic OR Fatty Livers, Nonalcoholic OR Liver, Nonalcoholic Fatty OR Livers, Nonalcoholic Fatty OR Nonalcoholic Fatty Liver OR Nonalcoholic Fatty Livers OR Nonalcoholic Steatohepatitis OR Nonalcoholic Steatohepatitides OR Steatohepatitides, Nonalcoholic OR Steatohepatitis, Nonalcoholic OR Non-alcoholic Fatty Liver Disease)

53 of Cochrane

Non alcoholic Fatty Liver Disease OR NAFLD OR Nonalcoholic Fatty Liver Disease OR Fatty Liver, Nonalcoholic OR Fatty Livers, Nonalcoholic OR Liver, Nonalcoholic Fatty OR Livers, Nonalcoholic Fatty OR Nonalcoholic Fatty Liver OR Nonalcoholic Fatty Livers OR Nonalcoholic Steatohepatitis OR Nonalcoholic Steatohepatitides OR Steatohepatitides, Nonalcoholic OR Steatohepatitis, Nonalcoholic OR Non-alcoholic Fatty Liver Disease in Title Abstract Keyword AND Gastrointestinal Microbiome OR Gastrointestinal Microbiomes OR Microbiome, Gastrointestinal OR Gut Microbiome OR Gut Microbiomes OR Microbiome, Gut OR Gut Microflora OR Microflora, Gut OR Gut Microbiota OR Gut Microbiotas OR Microbiota, Gut OR Gastrointestinal Flora OR Flora, Gastrointestinal OR Gut Flora OR Flora, Gut OR Gastrointestinal Microbiota OR Gastrointestinal Microbiotas OR Microbiota, Gastrointestinal OR Gastrointestinal Microbial Community OR Gastrointestinal Microbial Communities OR Microbial Community, Gastrointestinal OR Gastrointestinal Microflora OR Microflora, Gastrointestinal OR Gastric Microbiome OR Gastric Microbiomes OR Microbiome, Gastric OR Intestinal Microbiome OR Intestinal Microbiomes OR Microbiome, Intestinal OR Intestinal Microbiota OR Intestinal Microbiotas OR Microbiota, Intestinal OR Intestinal Microflora OR Microflora, Intestinal OR Intestinal Flora OR Flora, Intestinal OR Enteric Bacteria OR Bacteria, Enteric in Title Abstract Keyword AND Inflammation* OR Immune Activation OR Interleukin* OR Cytokine* OR Interferon OR Lymphocyte OR Macrophage OR Microglia OR Tumor Necrosis Factor-alpha OR C-Reactive Protein OR Transforming growth factor OR IFN OR IL OR CRP OR TGF OR TNF OR Inflammatory factor OR pro-inflammatory cytokine OR chemokine OR inflammatory cytokine OR YKL OR nuclear factor kappa OR hypoxia inducible factor OR hs-CRP OR interleukin-1 receptor antagonist OR E-selectin OR monocyte chemoattractant protein OR MCP OR intercellular adhesion molecule OR ICAM OR Interleukin receptor antagonist OR soluble adhesion molecules OR Chemokines in Title Abstract Keyword

**Appendix 1c**

186 of PubMed

((Probiotic[Title/Abstract] OR probiotic[Title/Abstract] OR symbiotic[Title/Abstract] OR symbiotic[Title/Abstract] OR symbiotic[Title/Abstract] OR lactobacill*[Title/Abstract] OR Lactobacillus[Title/Abstract] OR streptococcus[Title/Abstract] OR Streptococcus[Title/Abstract] OR bifidobacter[Title/Abstract] OR Bifidobacterium[Title/Abstract] OR saccharomyces[Title/Abstract] OR Pediococcus[Title/Abstract] OR Pediococcus[Title/Abstract])) AND (Non alcoholic Fatty Liver Disease[Title/Abstract] OR NAFLD[Title/Abstract] OR Nonalcoholic Fatty Liver Disease[Title/Abstract] OR Fatty Liver, Nonalcoholic[Title/Abstract] OR Fatty Livers, Nonalcoholic[Title/Abstract] OR Liver, Nonalcoholic Fatty[Title/Abstract] OR Livers, Nonalcoholic Fatty[Title/Abstract] OR Nonalcoholic Fatty Liver[Title/Abstract] OR Nonalcoholic Fatty Livers[Title/Abstract] OR Nonalcoholic Steatohepatitis[Title/Abstract] OR Nonalcoholic Steatohepatitides[Title/Abstract] OR Steatohepatitides, Nonalcoholic[Title/Abstract] OR Steatohepatitis, Nonalcoholic[Title/Abstract] OR Non-alcoholic Fatty Liver Disease[Title/Abstract])

318 of Embase

(probiotic:ab,ti OR symbiotic:ab,ti OR lactobacill*:ab,ti OR lactobacillus:ab,ti OR streptococcus:ab,ti OR bifidobacter:ab,ti OR bifidobacterium:ab,ti OR saccharomyces:ab,ti OR pediococcus:ab,ti) AND ('non alcoholic fatty liver disease':ab,ti OR nafld:ab,ti OR 'nonalcoholic fatty liver disease':ab,ti OR 'fatty liver, nonalcoholic':ab,ti OR 'fatty livers, nonalcoholic':ab,ti OR 'liver, nonalcoholic fatty':ab,ti OR 'livers, nonalcoholic fatty':ab,ti OR 'nonalcoholic fatty liver':ab,ti OR 'nonalcoholic fatty livers':ab,ti OR 'nonalcoholic steatohepatitis':ab,ti OR 'nonalcoholic steatohepatitides':ab,ti OR 'steatohepatitides, nonalcoholic':ab,ti OR 'steatohepatitis, nonalcoholic':ab,ti OR 'non-alcoholic fatty liver disease':ab,ti)

465 of Web of Science

TOPIC: (Probiotic OR probiotic OR symbiotic OR symbiotic OR symbiotic OR lactobacill* OR Lactobacillus OR streptococcus OR Streptococcus OR bifidobacter OR Bifidobacterium OR saccharomyces OR Pediococcus OR Pediococcus) AND TOPIC: (Non alcoholic Fatty Liver Disease OR NAFLD OR Nonalcoholic Fatty Liver Disease OR Fatty Liver, Nonalcoholic OR Fatty Livers, Nonalcoholic OR Liver, Nonalcoholic Fatty OR Livers, Nonalcoholic Fatty OR Nonalcoholic Fatty Liver OR Nonalcoholic Fatty Livers OR Nonalcoholic Steatohepatitis OR Nonalcoholic Steatohepatitides OR Steatohepatitides, Nonalcoholic OR Steatohepatitis, Nonalcoholic OR Non-alcoholic Fatty Liver Disease)

99 of Cochrane

Non alcoholic Fatty Liver Disease OR NAFLD OR Nonalcoholic Fatty Liver Disease OR Fatty Liver, Nonalcoholic OR Fatty Livers, Nonalcoholic OR Liver, Nonalcoholic Fatty OR Livers, Nonalcoholic Fatty OR Nonalcoholic Fatty Liver OR Nonalcoholic Fatty Livers OR Nonalcoholic Steatohepatitis OR Nonalcoholic Steatohepatitides OR Steatohepatitides, Nonalcoholic OR Steatohepatitis, Nonalcoholic OR Non-alcoholic Fatty Liver Disease in Title Abstract Keyword AND Probiotic OR probiotic OR symbiotic OR symbiotic OR symbiotic OR lactobacill* OR Lactobacillus OR streptococcus OR Streptococcus OR bifidobacter OR Bifidobacterium OR saccharomyces OR Pediococcus OR Pediococcus in Title Abstract Keyword

**Appendix 2:** Gut microbiota in NAFLD

|  | **Study** | **Subjects** | | **Phylum** | **Family** | **Genus** | |  |
| --- | --- | --- | --- | --- | --- | --- | --- | --- |
|  | Zhu 2013 [1] | NASH/obese vs. healthy children | | Actinobacteria↓Bacteroidetes↑Firmicutes↓Proteobacte-ria↑ | Bifidobacteriaceae↓Prevotellaceae↑Rikenellaceae↓Lachnospiraceae↓Ruminococcaceae↓ | Bifidobacterium↓Prevotella↑Alistipes ↓Blautia ↓Escherichia coli↑ | |  |
|  | Michail 2015 [2] | NAFLD children vs. healthy/Obese children with no NAFLD | | - | Gammaproteobacteria(class)↑ | Prevotella↑ | |  |
|  | Del 2017 [3] | Paediatric NAFLD, NASH,or obesity vs. healthy | | Actinobacteria↑Bacteroidetes↓ | Rikenellaceae↓ | Ruminococcus↑Blautia↑Dorea↑Bradyrhizobium↑Anaerococcus↑Peptoniphilus↑Propionibacterium acnes↑Oscillospira↓ | |  |
|  | Ling 2018 [4] | NAFLD children vs. healthy children with no NAFLD | | Actinomycetes↓ Thermus↑ | - | Bacteroides↓ Bifidobacterium↓Prevotella↑ | |  |
|  | Schwimmer 2019 [5] | NAFLD children vs. healthy children with no NAFLD | | Fusobacteria↑ Verrucomicrobia↑ and Lentisphaerae↑ | - | Lactobacillus↑Oribacterium ↑Oscillibacter↑ Lactonifactor↑ Akkermansia↑Enterococcus↑ | |  |
|  | Nobili 2016 [6] | NAFLD children vs. healthy children with no NAFLD | | - | - | Lactobacillus↑ L. mucosae↑ Bifidobacterium↓ | |  |
| Wong 2013 [7] | | | NASH patients vs. healthy subjects | - | - | Faecalibacterium↓Anaerosporobacter ↓Parabacteroides↑Allisonella↑ |  | |
| Raman 2013 [8] | | | NASH patients vs. healthy subjects | - | Lactobacillaceae↑Lachnospiraceae↑Ruminococcaceae ↓ | Lactobacillus↑Robinsoniella↑Roseburia ↑Dorea ↑Oscillibacter↓ |  | |
| Jiang 2015 [9] | | | NASH patients vs. healthy subjects | - | - | Alistipes↓Prevotella↓Escherichia coli↑Odoribacter↓Lactobacillus↑Oscillibacter↓Anaerobacter↑Clostridium XI↑Streptococcus↑Flavonifractor↓ |  | |
| Boursier 2016 [10] | | | NASH vs. no NASH | - | Bacteroidaceae↑Prevotellaceae↓ | Bacteroides↑Prevotella↓ |  | |

NAFLD, Nonalcoholic fatty liver disease; NASH, non-alcoholic steatohepatitis.

**Appendix 3: Gut microbial m**etabolites in NAFLD

|  | **Study** | **Material** | **Result** |
| --- | --- | --- | --- |
|  | Vrieze 2012[11] | Endotoxins | Gut bacterial-derived endotoxins may interact with pattern recognition receptors, including TLRs, which are expressed in various cells in the liver, including macrophages and kuppfer cells |
|  | Leavy 2015[12] | LPS | The complex formed by LPS and LBP binds with CD14 to activate the innate immune recognition system |
|  | Fernandes 2014[13] | SCFAs,butyrate, propionate | Obese subjects have higher SCFA products in stool samples than lean subjects because of the differences in their colonic fermentation. The SCFA main products display different mechanisms to induce satiety: butyrate acts on intestinal cells thereby increasing glucagon-like peptide-1 (GLP-1) production, and propionate affects intestinal gluconeogenesis, both pathways leading to an amelioration of glucose homeostasis and satiety enhancement. |
|  | Pappo 1992[14] | LPS | Previous study has demonstrated that treatment with antibiotics (e.g., polymyxin B) targeting gram-negative bacteria efficiently reduced tumor necrosis factor (TNF) production and plasma LPS levels, leading to the reversal of hepatic steatosis. |
|  | Guo 2015[15] | LPS | The mechanism by which LPS produced by gut microbiota contributes to the occurrence and development of NAFLD involves the intestinal barrier dysfunction. Several lines of evidence have shown that increments of circulating LPS impairs intestinal barrier function and causes subsequent increases in intestinal permeability. This occurs through the TLR-4-dependent up-regulation of CD14 and MLCK (Myosin light chain kinase), and activation of IRAK-4 (IL-1R-associated kinase 4). |
|  | Ehses 2010[16] | Peptidoglycan (PGN) | The role of TLR2 and Peptidoglycan (PGN) in the pathophysiology of NASH has been controversial depending on the model of NASH. In high fat diet-induced obese mice, TLR2 deficiency has been shown to be resistant to insulin resistance, hepatic steatosis, and tissue inflammation. |
|  | Choi 2011 [17] | Extracellular vesicles (EVs) | As innate immune is a key mechanism mediating the inflammation and other pathological progress in NAFLD, EVs may regulate NAFLD through transferring the contents into Kupffer cells, stellate cells, and hepatocytes. |
|  | Beaumont 2018 [18] | Indole | Mice receiving indole display resistance to liver inflammation and metabolic alternations of cholesterol induced by LPS. This observation suggests that indole may improve inflammatory disorder in the liver. Other indole derivatives that have received widespread attentions include indole-3-aldehyde (IAld), indole-3-acetic acid (IAA), and indole-3-propionic acid (IPA). |
|  | Wahlstrom 2016 [19] | Bile Acids | Gut microbiota converts the primary bile acids including cholic acid (CA) and chenodeoxycholic acid (CDCA) in the distal small intestine and colon of human beings into secondary bile acids such as deoxycholic acid (DCA), lithocholic acid (LCA), and ursodeoxycholic acid (UDCA). |
| Zhao 2013 [20] | | SCFAs | Interestingly, the number of Bacteroidetes was associated with lower levels of certain fecal metabolites such as amino acids and short-chain fatty acids(SCFAs). |
| Sasaki 2011 [21] | | Indole | Numerous bacterial species including the genera of Prevotella, Bacteroides, Fusobacterium, and Escherichia possess the capacity to degrade tryptophan into indole by tryptophanase. |
| Warrier 2015 [22] | | Trimethylamine-N-oxide (TMAO) | TMAO may modulate NAFLD via the regulation of bile acid metabolism and transport. Finally, gut microbiota-mediated TMA/FMO3/TMAO pathway modulates insulin resistance, glycolipid metabolism, cholesterol homeostasis, and hepatic inflammation, thereby affecting hepatic triglyceride accumulation and liver steatosis. |

NAFLD, Nonalcoholic fatty liver disease; NASH, non-alcoholic steatohepatitis; LPS, lipopolysaccharides; SCFAs, Short-chain fatty acids;

**Appendix 4:** Characteristics of included studies for inflammatory factors and probiotics treatment

| **Study** | **Type of study** | **Sample size** | **Country** | **Mean age** | **Follow up time** | **NAFLD type** | **Diagnosis of NAFLD** |
| --- | --- | --- | --- | --- | --- | --- | --- |
| Aller 2011 [23] | Double blind randomized controlled trial | 30 | Spain | 49.4±10.9 | 3 months | NAFLD | Liver biopsy |
| Asgharian 2016 [24] | Randomized,double-blind,placebo-controlled clinical trial | 80 | Iran | 46.6±1.7 | 8 weeks | NAFLD | Ultrasonography |
| Ekhlasi 2017 [25] | Randomized,double-blind,placebo-controlled trial | 60 | Iran | 44.0±20.0 | 8 weeks | NAFLD | Ultrasonography |
| Eslamparast 2014 [26] | Randomized, double-blind, placebo-controlled clinical trial | 52 | Iran | 46.0±9.2 | 28 weeks | NAFLD | Ultrasonography |
| Loguercio 2002 [27] | Clinical trial | 10 | Italy | 37.0±2.7 | 2 months | NASH | Liver biopsy |
| Malaguarnera 2012 [28] | Randomized, double-blind, placebo-controlled clinical trial | 75 | Italy | 46.9±5.4 | 24 weeks | NASH | Liver biopsy |
| Mofidi 2017 [29] | Randomized, double-blind, placebo-controlled clinical trial | 50 | Iran | 45.4±10.7 | 28 weeks | NAFLD | Ultrasonography |
| Mykhal’chyshyn 2013 [30] | Clinical trial | 72 | Ukrainian | 35.5±10.9 | 1 months | NAFLD | Ultrasonography |
| Sepideh 2016 [31] | Double-blind, randomized clinical trial | 42 | Iran | 44.7±1.6 | 8 weeks | NAFLD | Ultrasonography |
| Sherf-Dagan 2018 [32] | Randomized, double-blind, placebo-controlled clinical trial | 100 | Israel | 41.9±9.8 | 6 months | NAFLD | Liver biopsy |
| Vajro 2011[33] | Randomized, double-blind, placebo-controlled clinical trial | 20 | Italy | 10.7±2.1 | 8 weeks | NAFLD | Ultrasonography |
| Wang 2018[34] | Randomized, double-blind, placebo-controlled clinical trial | 200 | China | 42.9±5.0 | 4 weeks | NAFLD | Ultrasonography |
| Yang 2012 [35] | Randomized, double-blind, placebo-controlled clinical trial | 60 | China | 47.5±12.3 | 4 weeks | NASH | Liver biopsy |

NAFLD, Nonalcoholic fatty liver disease; NASH, non-alcoholic steatohepatitis.

**Appendix 5: Egger funnel plot for the TNF-α in NAFLD group**


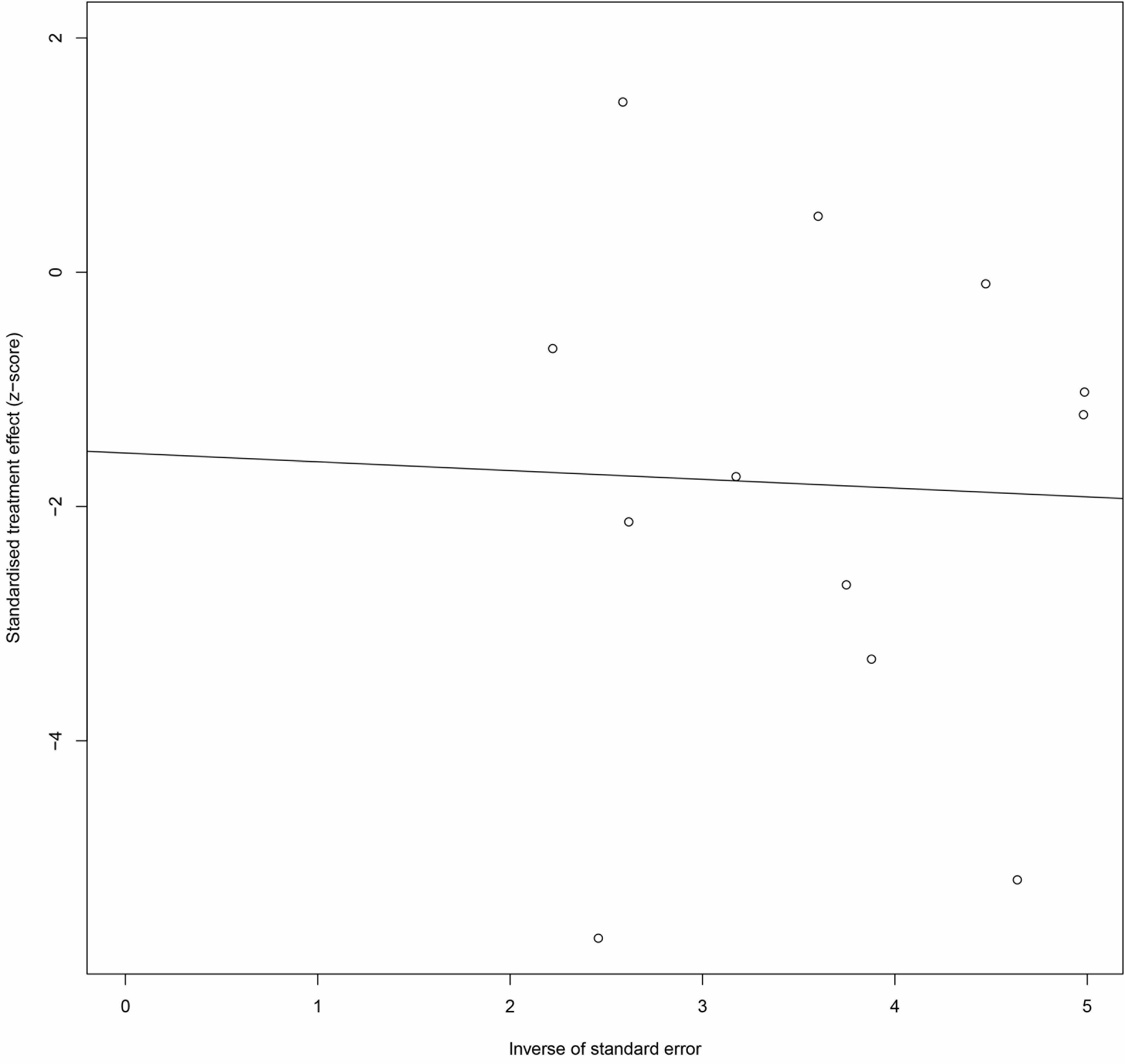


t = -0.6125, df = 10, p-value = 0.5539

**Appendix 6: Risk of bias assessments**


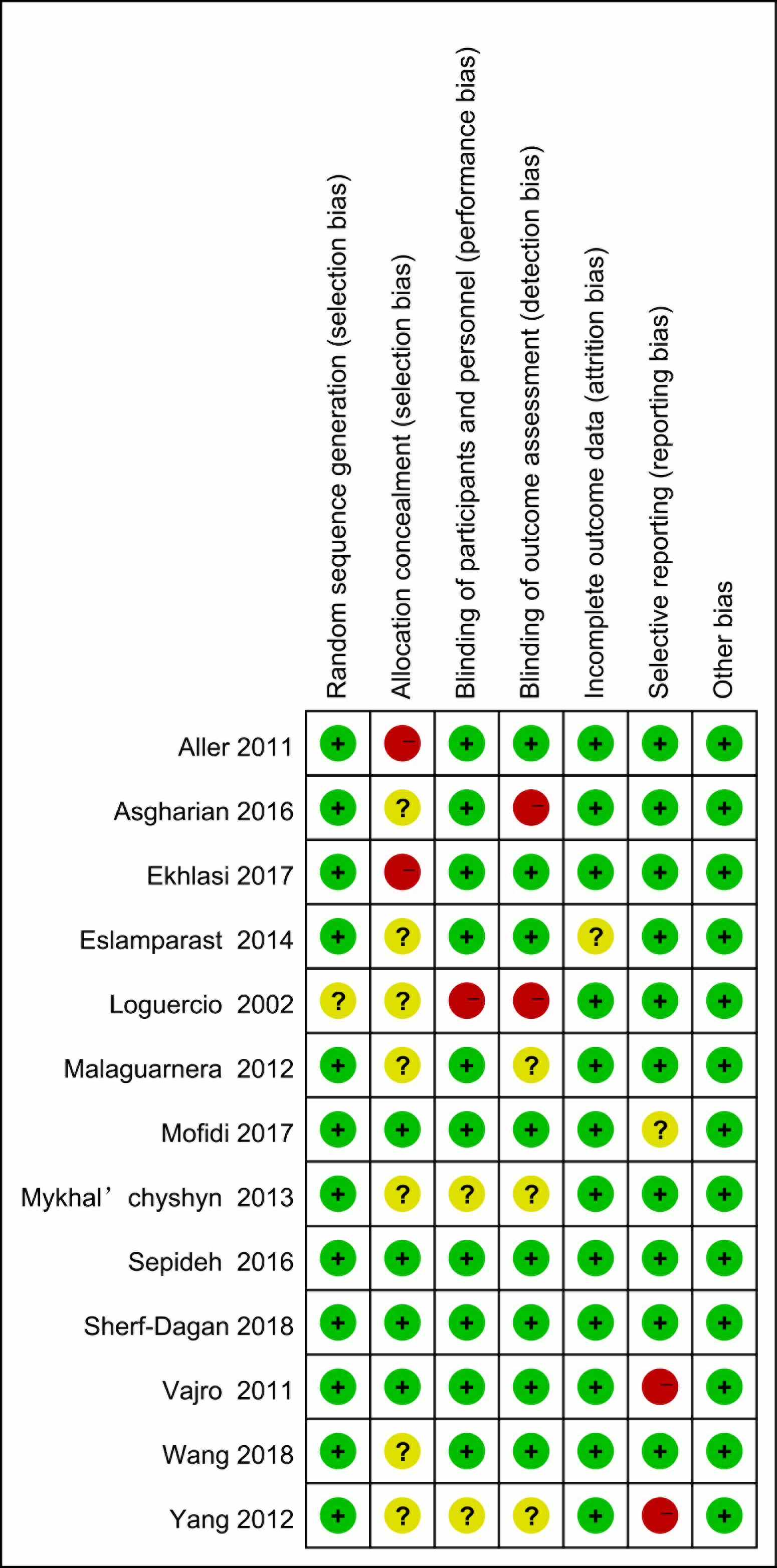

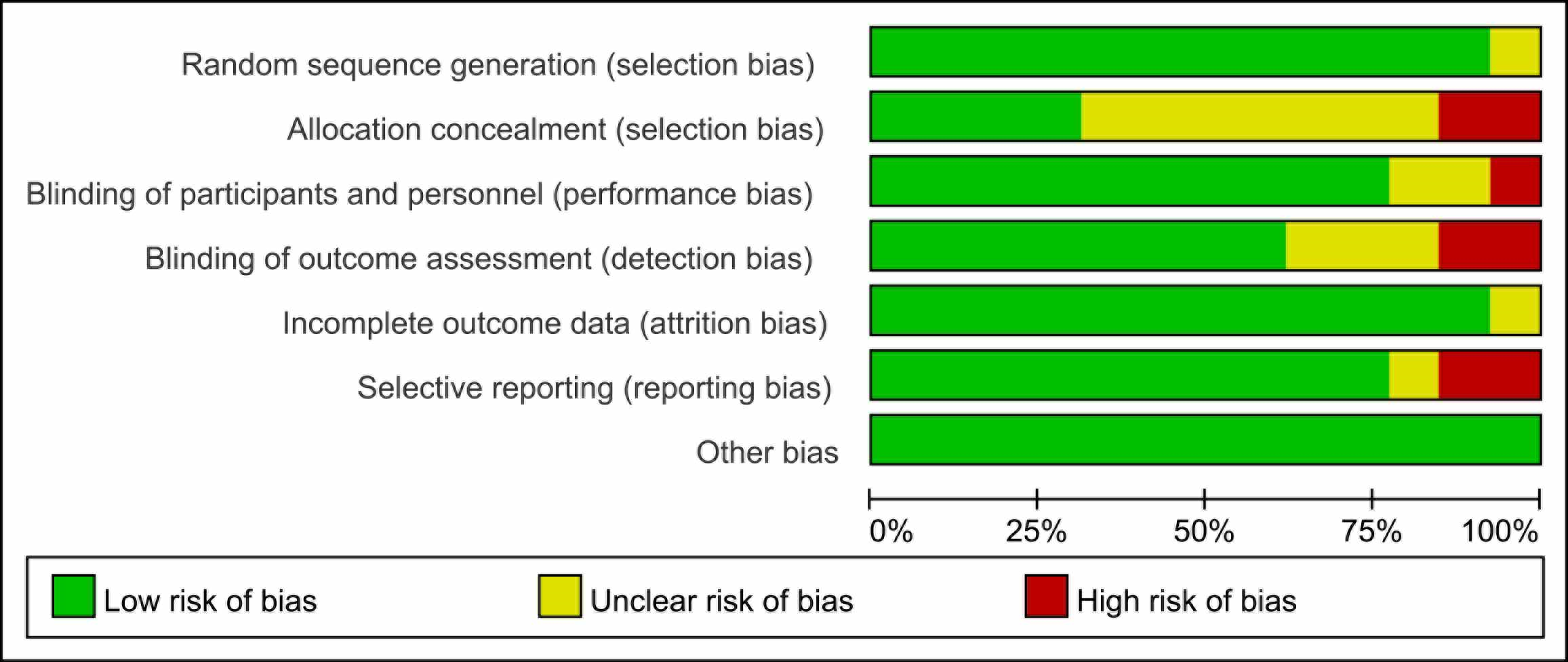


1. Zhu L, Baker SS, Gill C, Liu W, Alkhouri R, Baker RD, Gill SR: **Characterization of gut microbiomes in nonalcoholic steatohepatitis (NASH) patients: a connection between endogenous alcohol and NASH**. *Hepatology* 2013, **57**(2):601-609.

2. Michail S, Lin M, Frey MR, Fanter R, Paliy O, Hilbush B, Reo NV: **Altered gut microbial energy and metabolism in children with non-alcoholic fatty liver disease**. *FEMS Microbiol Ecol* 2015, **91**(2):1-9.

3. Del Chierico F, Nobili V, Vernocchi P, Russo A, De Stefanis C, Gnani D, Furlanello C, Zandona A, Paci P, Capuani G *et al*: **Gut microbiota profiling of pediatric nonalcoholic fatty liver disease and obese patients unveiled by an integrated meta-omics-based approach**. *Hepatology* 2017, **65**(2):451-464.

4. Ling JR, Zhang YJ, Zhang ZH, He BH, Ni W, Shi WQ, Chen ZY: **[Specific changes of intestinal microflora in children with nonalcoholic fatty liver disease]**. *Zhonghua er ke za zhi = Chinese journal of pediatrics* 2018, **56**(11):850-855.

5. Schwimmer JB, Johnson JS, Angeles JE, Behling C, Belt PH, Borecki I, Bross C, Durelle J, Goyal NP, Hamilton G *et al*: **Microbiome Signatures Associated With Steatohepatitis and Moderate to Severe Fibrosis in Children With Nonalcoholic Fatty Liver Disease**. *Gastroenterology* 2019.

6. Nobili V, Putignani L, Mosca A, Chierico FD, Vernocchi P, Alisi A, Stronati L, Cucchiara S, Toscano M, Drago L: **Bifidobacteria and lactobacilli in the gut microbiome of children with non-alcoholic fatty liver disease: which strains act as health players?** *Arch Med Sci* 2018, **14**(1):81-87.

7. Wong VW, Won GL, Chim AM, Chu WC, Yeung DK, Li KC, Chan HL: **Treatment of nonalcoholic steatohepatitis with probiotics. A proof-of-concept study**. *Ann Hepatol* 2013, **12**(2):256-262.

8. Raman M, Ahmed I, Gillevet PM, Probert CS, Ratcliffe NM, Smith S, Greenwood R, Sikaroodi M, Lam V, Crotty P *et al*: **Fecal microbiome and volatile organic compound metabolome in obese humans with nonalcoholic fatty liver disease**. *Clin Gastroenterol Hepatol* 2013, **11**(7):868-875.e861-863.

9. Jiang C, Xie C, Li F, Zhang L, Nichols RG, Krausz KW, Cai J, Qi Y, Fang ZZ, Takahashi S *et al*: **Intestinal farnesoid X receptor signaling promotes nonalcoholic fatty liver disease**. *J Clin Invest* 2015, **125**(1):386-402.

10. Boursier J, Mueller O, Barret M, Machado M, Fizanne L, Araujo-Perez F, Guy CD, Seed PC, Rawls JF, David LA *et al*: **The severity of nonalcoholic fatty liver disease is associated with gut dysbiosis and shift in the metabolic function of the gut microbiota**. *Hepatology* 2016, **63**(3):764-775.

11. Vrieze A, Van Nood E, Holleman F, Salojarvi J, Kootte RS, Bartelsman JF, Dallinga-Thie GM, Ackermans MT, Serlie MJ, Oozeer R *et al*: **Transfer of intestinal microbiota from lean donors increases insulin sensitivity in individuals with metabolic syndrome**. *Gastroenterology* 2012, **143**(4):913-916.e917.

12. Leavy O: **Innate immunity: New PAMP discovered**. *Nature reviews Immunology* 2015, **15**(7):402-403.

13. Fernandes J, Su W, Rahat-Rozenbloom S, Wolever TM, Comelli EM: **Adiposity, gut microbiota and faecal short chain fatty acids are linked in adult humans**. *Nutr Diabetes* 2014, **4**:e121.

14. Pappo I, Bercovier H, Berry EM, Haviv Y, Gallily R, Freund HR: **Polymyxin B reduces total parenteral nutrition-associated hepatic steatosis by its antibacterial activity and by blocking deleterious effects of lipopolysaccharide**. *JPEN Journal of parenteral and enteral nutrition* 1992, **16**(6):529-532.

15. Guo S, Nighot M, Al-Sadi R, Alhmoud T, Nighot P, Ma TY: **Lipopolysaccharide Regulation of Intestinal Tight Junction Permeability Is Mediated by TLR4 Signal Transduction Pathway Activation of FAK and MyD88**. *J Immunol* 2015, **195**(10):4999-5010.

16. Ehses JA, Meier DT, Wueest S, Rytka J, Boller S, Wielinga PY, Schraenen A, Lemaire K, Debray S, Van Lommel L *et al*: **Toll-like receptor 2-deficient mice are protected from insulin resistance and beta cell dysfunction induced by a high-fat diet**. *Diabetologia* 2010, **53**(8):1795-1806.

17. Choi DS, Kim DK, Choi SJ, Lee J, Choi JP, Rho S, Park SH, Kim YK, Hwang D, Gho YS: **Proteomic analysis of outer membrane vesicles derived from Pseudomonas aeruginosa**. *Proteomics* 2011, **11**(16):3424-3429.

18. Beaumont M, Neyrinck AM, Olivares M, Rodriguez J, de Rocca Serra A, Roumain M, Bindels LB, Cani PD, Evenepoel P, Muccioli GG *et al*: **The gut microbiota metabolite indole alleviates liver inflammation in mice**. *Faseb j* 2018:fj201800544.

19. Wahlstrom A, Sayin SI, Marschall HU, Backhed F: **Intestinal Crosstalk between Bile Acids and Microbiota and Its Impact on Host Metabolism**. *Cell Metab* 2016, **24**(1):41-50.

20. Zhao Y, Wu J, Li JV, Zhou NY, Tang H, Wang Y: **Gut microbiota composition modifies fecal metabolic profiles in mice**. *J Proteome Res* 2013, **12**(6):2987-2999.

21. Sasaki-Imamura T, Yoshida Y, Suwabe K, Yoshimura F, Kato H: **Molecular basis of indole production catalyzed by tryptophanase in the genus Prevotella**. *FEMS Microbiol Lett* 2011, **322**(1):51-59.

22. Warrier M, Shih DM, Burrows AC, Ferguson D, Gromovsky AD, Brown AL, Marshall S, McDaniel A, Schugar RC, Wang Z *et al*: **The TMAO-Generating Enzyme Flavin Monooxygenase 3 Is a Central Regulator of Cholesterol Balance**. *Cell Rep* 2015, **10**(3):326-338.

23. Aller R, De Luis DA, Izaola O, Conde R, Gonzalez Sagrado M, Primo D, De La Fuente B, Gonzalez J: **Effect of a probiotic on liver aminotransferases in nonalcoholic fatty liver disease patients: a double blind randomized clinical trial**. *Eur Rev Med Pharmacol Sci* 2011, **15**(9):1090-1095.

24. Asgharian A, Askari G, Esmailzade A, Feizi A, Mohammadi V: **The Effect of Symbiotic Supplementation on Liver Enzymes, C-reactive Protein and Ultrasound Findings in Patients with Non-alcoholic Fatty Liver Disease: A Clinical Trial**. *International Journal of Preventive Medicine* 2016, **7**(1):59.

25. Ekhlasi G, Zarrati M, Agah S, Hosseini AF, Hosseini S, Shidfar S, Soltani Aarbshahi SS, Razmpoosh E, Shidfar F: **Effects of symbiotic and vitamin E supplementation on blood pressure, nitric oxide and inflammatory factors in non-alcoholic fatty liver disease**. *Excli j* 2017, **16**:278-290.

26. Eslamparast T, Poustchi H, Zamani F, Sharafkhah M, Malekzadeh R, Hekmatdoost A: **Synbiotic supplementation in nonalcoholic fatty liver disease: a randomized, double-blind, placebo-controlled pilot study**. *Am J Clin Nutr* 2014, **99**(3):535-542.

27. Loguercio C, De Simone T, Federico A, Terracciano F, Tuccillo C, Di Chicco M, Carteni M: **Gut-liver axis: a new point of attack to treat chronic liver damage?** *Am J Gastroenterol* 2002, **97**(8):2144-2146.

28. Malaguarnera M, Vacante M, Antic T, Giordano M, Chisari G, Acquaviva R, Mastrojeni S, Malaguarnera G, Mistretta A, Li Volti G *et al*: **Bifidobacterium longum with fructo-oligosaccharides in patients with non alcoholic steatohepatitis**. *Dig Dis Sci* 2012, **57**(2):545-553.

29. Mofidi F, Poustchi H, Yari Z, Nourinayyer B, Merat S, Sharafkhah M, Malekzadeh R, Hekmatdoost A: **Synbiotic supplementation in lean patients with non-alcoholic fatty liver disease: a pilot, randomised, double-blind, placebo-controlled, clinical trial**. *Br J Nutr* 2017, **117**(5):662-668.

30. Mykhal'chyshyn HP, Bodnar PM, Kobyliak NM: **[Effect of probiotics on proinflammatory cytokines level in patients with type 2 diabetes and nonalcoholic fatty liver disease]**. *Lik Sprava* 2013(2):56-62.

31. Sepideh A, Karim P, Hossein A, Leila R, Hamdollah M, Mohammad EG, Mojtaba S, Mohammad S, Ghader G, Seyed Moayed A: **Effects of Multistrain Probiotic Supplementation on Glycemic and Inflammatory Indices in Patients with Nonalcoholic Fatty Liver Disease: A Double-Blind Randomized Clinical Trial**. *Journal of the American College of Nutrition* 2016, **35**(6):500-505.

32. Sherf-Dagan S, Zelber-Sagi S, Zilberman-Schapira G, Webb M, Buch A, Keidar A, Raziel A, Sakran N, Goitein D, Goldenberg N *et al*: **Probiotics administration following sleeve gastrectomy surgery: a randomized double-blind trial**. *International journal of obesity (2005)* 2018, **42**(2):147-155.

33. Vajro P, Mandato C, Licenziati MR, Franzese A, Vitale DF, Lenta S, Caropreso M, Vallone G, Meli R: **Effects of Lactobacillus rhamnosus strain GG in pediatric obesity-related liver disease**. *Journal of pediatric gastroenterology and nutrition* 2011, **52**(6):740-743.

34. Wang W, Shi LP, Shi L, Xu L: **[Efficacy of probiotics on the treatment of non-alcoholic fatty liver disease]**. *Zhonghua nei ke za zhi* 2018, **57**(2):101.

35. Yang LH, Guo H, Cai J, Cai XW, Liu GL, Gastroenterology DO: **Intervention effect of microbiological capsules containing Bacillus subtilis and Enterococcus on intestinal flora in patients with NASH**. *World Chinese Journal of Digestology* 2012, **20**(20):1873.
